# Supplementary figures and images for: Development of emotional labor ability scale for kindergarten teachers
Source: PLoS One. 2025 Jun 23;20(6):e0325891. doi: 10.1371/journal.pone.0325891 (PMC12184924; doi:10.1371/journal.pone.0325891)

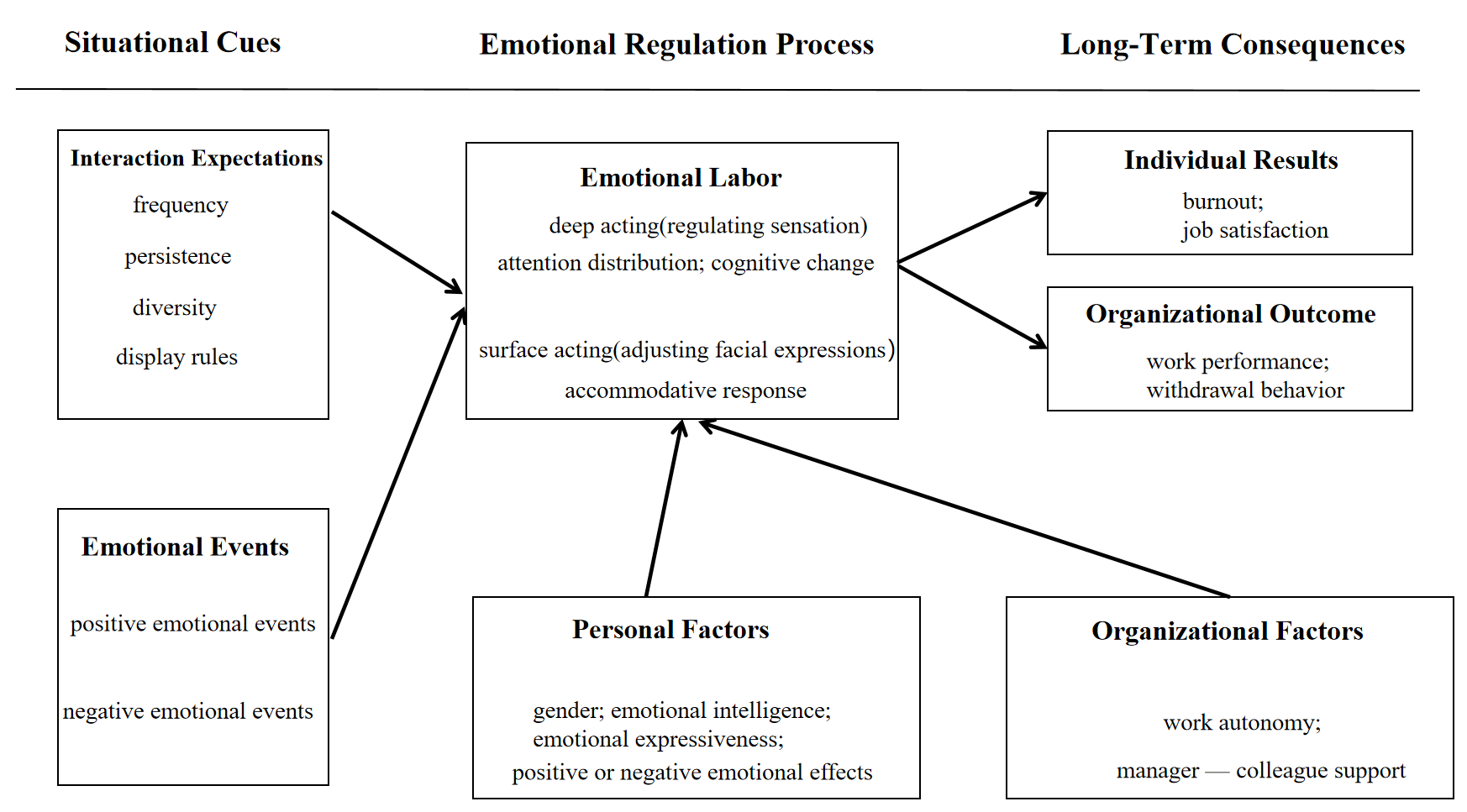


Working Mechanism of Emotional Labor

Supplement: S1 Fig — (DOCX) [file pone.0325891.s001.docx]

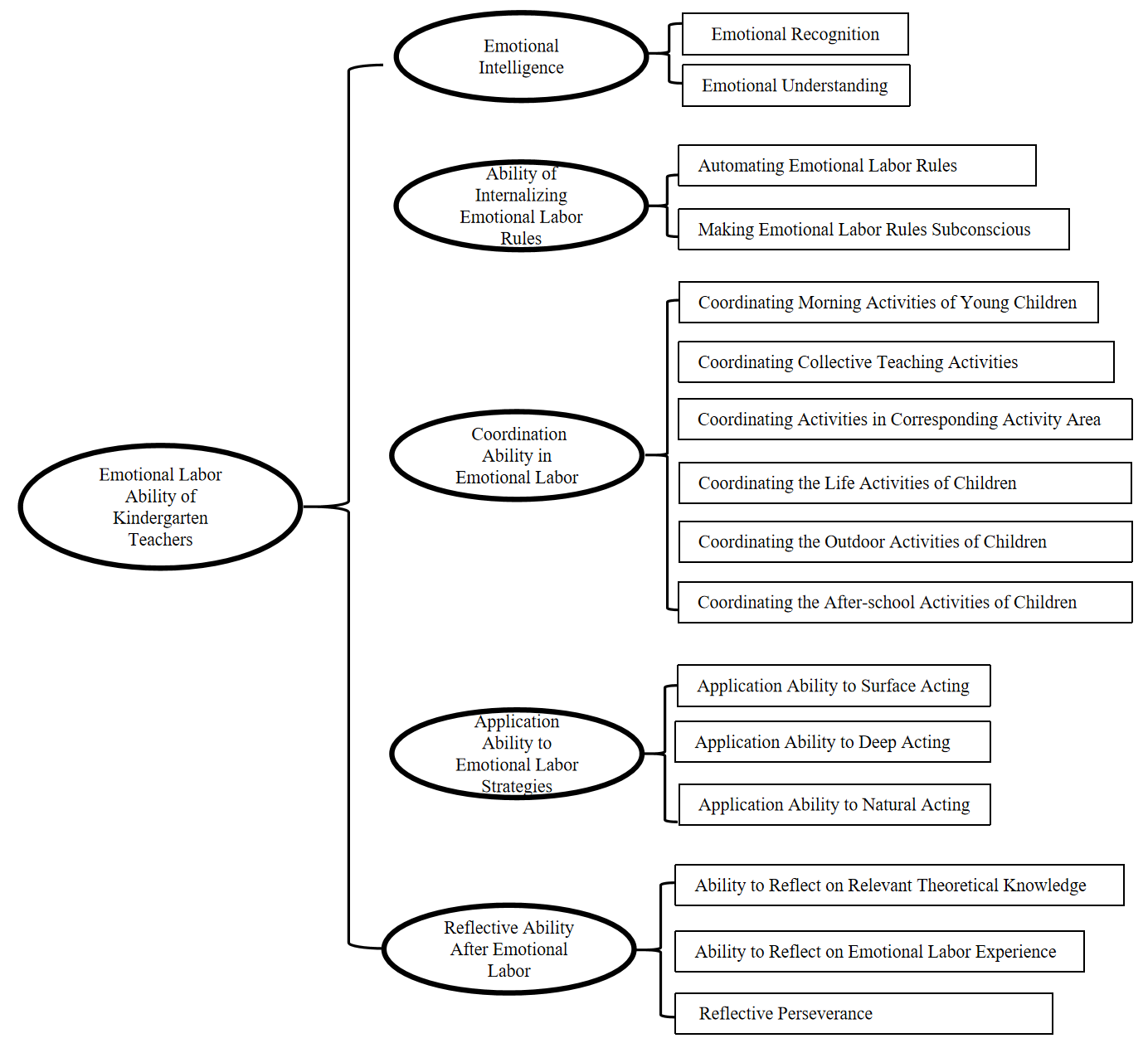


Figure 2 The Initial Model for the Emotional Labor Ability of Kindergarten Teachers

Supplement: S2 Fig — (DOCX) [file pone.0325891.s002.docx]

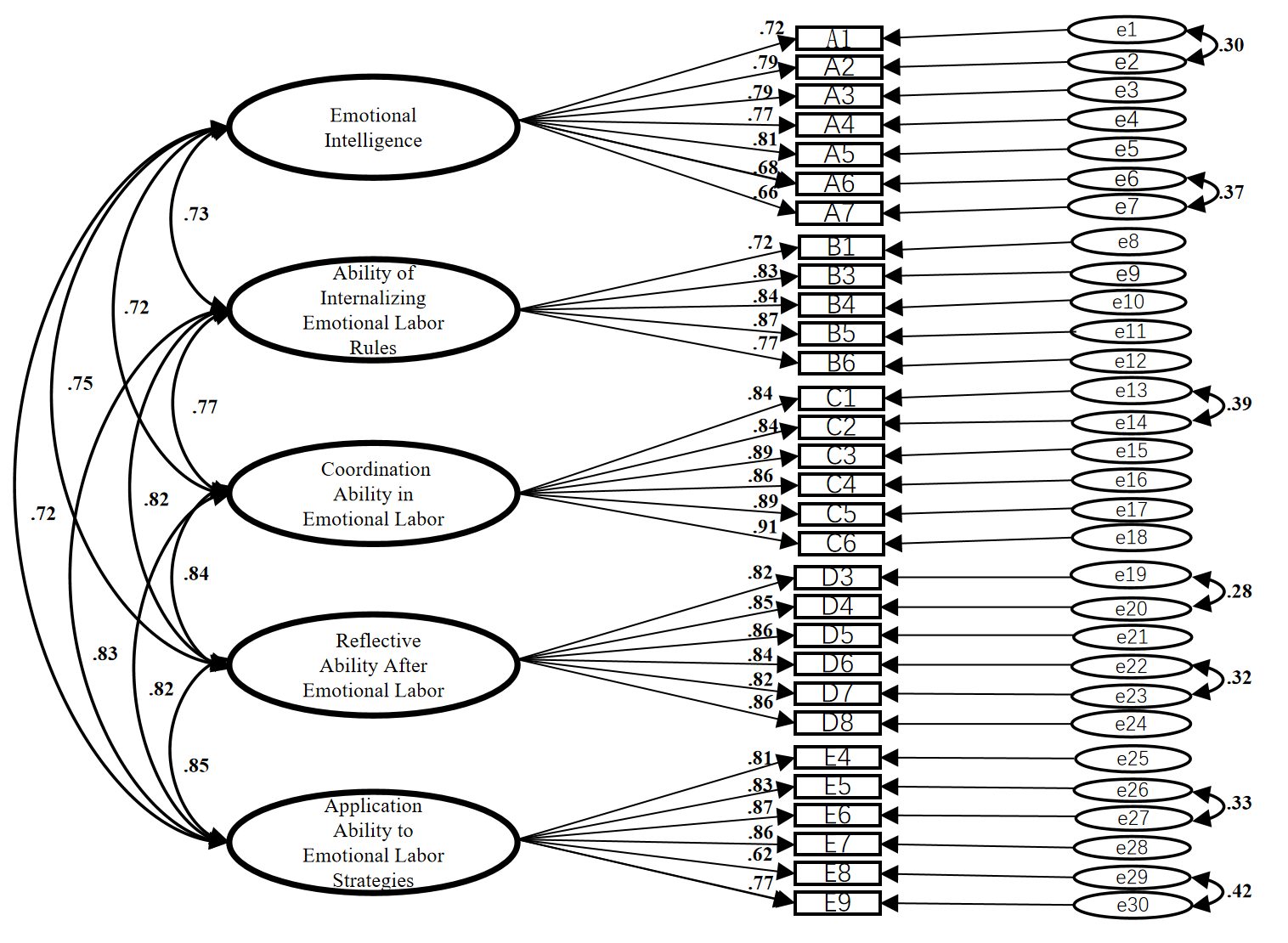


Figure 3 The Final Model of the Emotional Labor Ability of Kindergarten Teachers

Supplement: S3 Fig — (DOCX) [file pone.0325891.s003.docx]
