# Supplementary material for: Development of emotional labor ability scale for kindergarten teachers
Source: PLoS One. 2025 Jun 23;20(6):e0325891. doi: 10.1371/journal.pone.0325891 (PMC12184924; doi:10.1371/journal.pone.0325891)
Supplement: S1 Table — (DOCX) [file pone.0325891.s004.docx]

| Table 1 Demographic Characteristics of the Experts | | |
| --- | --- | --- |
| Characteristics | Frequency (N) | Proportion (%) |
| **Age (years)** |  |  |
| 30-40 | 1 | 20% |
| 40-50 | 3 | 60% |
| ≥50 | 1 | 20% |
| **Highest educational degree** |  |  |
| College | 0 | 0% |
| Undergraduate | 1 | 20% |
| Master’s and doctorate | 4 | 80% |
| **Title** |  |  |
| Junior professional title | 1 | 20% |
| Senior professional title | 2 | 40% |
| Associate senior professional title | 2 | 40% |
| **Work experience (years)** |  |  |
| 11–15 | 3 | 60% |
| 15–20 | 1 | 20% |
| ≥20 | 1 | 20% |
| **Research field** |  |  |
| Kindergarten teacher education | 2 | 40% |
| Kindergarten Teacher Management | 2 | 40% |
| Basic Theory of Preschool Education | 1 | 20% |
