# Supplementary material for: Development of emotional labor ability scale for kindergarten teachers
Source: PLoS One. 2025 Jun 23;20(6):e0325891. doi: 10.1371/journal.pone.0325891 (PMC12184924; doi:10.1371/journal.pone.0325891)
Supplement: S2 Table — (DOCX) [file pone.0325891.s005.docx]

| Table2 Correlation Coefficients Between Items and Total Score (N=409) | | | | | |
| --- | --- | --- | --- | --- | --- |
| Item | Low Group (N=111, M±SD) | High Group (N=111, M±SD) | C.R. Value | Correlation Coefficients (N=409) | α if Deleted |
| A1 | 3.72±0.80 | 4.39±0.59 | -7.07*** | 0.63*** | 0.97 |
| A2 | 3.63±0.79 | 4.49±0.59 | -9.20*** | 0.68*** | 0.97 |
| A3 | 3.54±0.84 | 4.39±0.58 | -8.77*** | 0.71*** | 0.97 |
| A4 | 3.45±0.85 | 4.40±0.61 | -9.54*** | 0.67*** | 0.97 |
| A5 | 3.65±0.81 | 4.48±0.59 | -8.77*** | 0.69*** | 0.97 |
| A6 | 3.51±0.81 | 4.41±0.61 | -9.29*** | 0.66*** | 0.97 |
| A7 | 3.32±0.81 | 4.38±0.60 | -10.98*** | 0.70*** | 0.97 |
| A8 | 3.45±0.75 | 4.39±0.61 | -10.26*** | 0.66*** | 0.97 |
| B1 | 3.44±0.83 | 4.70±0.50 | -13.76*** | 0.72*** | 0.97 |
| B2 | 3.23±0.84 | 4.44±0.64 | -12.01*** | 0.63*** | 0.97 |
| B3 | 3.67±0.89 | 4.71±0.46 | -11.04*** | 0.74*** | 0.97 |
| B4 | 3.58±0.73 | 4.62±0.51 | -12.37*** | 0.75*** | 0.97 |
| B5 | 3.50±0.87 | 4.70±0.46 | -12.80*** | 0.76*** | 0.97 |
| B6 | 3.34±0.99 | 4.63±0.50 | -12.26*** | 0.68*** | 0.97 |
| E1 | 3.19±0.79 | 4.46±0.67 | -12.89*** | 0.66*** | 0.97 |
| E2 | 3.29±0.82 | 4.42±0.73 | -10.84*** | 0.63*** | 0.97 |
| E3 | 3.26±0.83 | 4.42±0.70 | -11.33*** | 0.65*** | 0.97 |
| E4 | 3.44±0.88 | 4.70±0.46 | -13.38*** | 0.72*** | 0.97 |
| E5 | 3.38±0.89 | 4.76±0.47 | -14.49*** | 0.77*** | 0.97 |
| E6 | 3.32±0.83 | 4.77±0.45 | -16.07*** | 0.79*** | 0.97 |
| E7 | 3.58±0.80 | 4.76±0.47 | -13.34*** | 0.81*** | 0.97 |
| E8 | 3.10±0.92 | 4.40±0.97 | -10.23*** | 0.57*** | 0.97 |
| E9 | 3.39±0.86 | 4.68±0.56 | -13.29*** | 0.74*** | 0.97 |
| C1 | 3.51±0.84 | 4.59±0.53 | -11.36*** | 0.79*** | 0.97 |
| C2 | 3.53±0.75 | 4.58±0.61 | -11.39*** | 0.75*** | 0.97 |
| C3 | 3.61±0.77 | 4.67±0.51 | -12.08*** | 0.79*** | 0.97 |
| C4 | 3.59±0.73 | 4.71±0.48 | -13.60*** | 0.76*** | 0.97 |
| C5 | 3.49±0.85 | 4.72±0.45 | -13.50*** | 0.81*** | 0.97 |
| C6 | 3.59±0.83 | 4.68±0.49 | -12.09*** | 0.80*** | 0.97 |
| D1 | 3.53±0.85 | 4.68±0.51 | -12.17*** | 0.78*** | 0.97 |
| D2 | 3.58±0.83 | 4.68±0.56 | -11.73*** | 0.78*** | 0.97 |
| D3 | 3.50±0.80 | 4.60±0.51 | -12.35*** | 0.77*** | 0.97 |
| D4 | 3.43±0.77 | 4.61±0.51 | -13.48*** | 0.78*** | 0.97 |
| D5 | 3.50±0.74 | 4.59±0.51 | -12.80*** | 0.80*** | 0.97 |
| D6 | 3.43±0.86 | 4.59±0.51 | -12.24*** | 0.78*** | 0.97 |
| D7 | 3.38±0.79 | 4.56±0.57 | -12.82*** | 0.77*** | 0.97 |
| D8 | 3.30±0.82 | 4.56±0.53 | -13.63*** | 0.80*** | 0.97 |
| Notes: *p<0.05,**p<0.01, ***p<0.001. | | | | | |
| Abbreviations: C.R. Value, critical ratio value. | | | | | |
