# Supplementary material for: Development of emotional labor ability scale for kindergarten teachers
Source: PLoS One. 2025 Jun 23;20(6):e0325891. doi: 10.1371/journal.pone.0325891 (PMC12184924; doi:10.1371/journal.pone.0325891)
Supplement: S3 Table — (DOCX) [file pone.0325891.s006.docx]

| Table 3 Exploratory Factor Analysis Results of the Questionnaire of the Emotional Labor Ability of Kindergarten Teachers | | | | | | |
| --- | --- | --- | --- | --- | --- | --- |
| Item | Factor Loading | | | | | Commonality |
|  | 1 | 2 | 3 | 4 | 5 |  |
| A2 | .737 |  |  |  |  | .703 |
| A1 | .731 |  |  |  |  | .664 |
| A5 | .668 |  |  |  |  | .684 |
| A4 | .640 |  |  |  |  | .619 |
| A3 | .626 |  |  |  |  | .629 |
| A6 | .616 |  |  |  |  | .625 |
| A8 | .605 |  |  |  |  | .621 |
| A7 | .581 |  |  |  |  | .637 |
| C3 |  | .779 |  |  |  | .845 |
| C2 |  | .756 |  |  |  | .779 |
| C1 |  | .744 |  |  |  | .814 |
| C4 |  | .725 |  |  |  | .765 |
| C6 |  | .707 |  |  |  | .809 |
| C5 |  | .654 |  |  |  | .771 |
| D6 |  |  | .726 |  |  | .788 |
| D7 |  |  | .704 |  |  | .759 |
| D8 |  |  | .661 |  |  | .757 |
| D4 |  |  | .652 |  |  | .738 |
| D5 |  |  | .636 |  |  | .746 |
| D3 |  |  | .602 |  |  | .694 |
| D2 |  |  | .596 |  |  | .713 |
| D1 |  |  | .560 |  |  | .717 |
| E8 |  |  |  | .722 |  | .620 |
| E6 |  |  |  | .679 |  | .781 |
| E5 |  |  |  | .666 |  | .751 |
| E9 |  |  |  | .647 |  | .713 |
| E4 |  |  |  | .632 |  | .663 |
| E7 |  |  |  | .607 |  | .762 |
| B6 |  |  |  |  | .736 | .715 |
| B5 |  |  |  |  | .705 | .780 |
| B3 |  |  |  |  | .658 | .718 |
| B4 |  |  |  |  | .605 | .680 |
| B1 |  |  |  |  | .602 | .619 |
| Eigenvalue | 18.337 | 1.592 | 1.464 | 1.228 | 1.058 | Total |
| Total variance explained | 55.567% | 4.825% | 4.437% | 3.722% | 3.206% | 71.757% |
