# Supplementary material for: Development of emotional labor ability scale for kindergarten teachers
Source: PLoS One. 2025 Jun 23;20(6):e0325891. doi: 10.1371/journal.pone.0325891 (PMC12184924; doi:10.1371/journal.pone.0325891)
Supplement: S4 Table — (DOCX) [file pone.0325891.s007.docx]

| Table 4 Fitting Indicator of the Model of the Emotional Labor Ability of Teachers | | | | | | | |
| --- | --- | --- | --- | --- | --- | --- | --- |
| Fitting indicator | χ^2^/df | RMR | GFI | IFI | TLI | CFI | RMSEA |
| Recommended value | <3 | <0.05 | >0.90 | >0.90 | >0.90 | >0.90 | <0.08 |
| Fitted value of one-factor model | 9.099 | 0.027 | 0.720 | 0.853 | 0.839 | 0.853 | 0.100 |
| Fitted value of five-factor model | 2.774 | 0.016 | 0.918 | 0.969 | 0.965 | 0.969 | 0.047 |
