# Supplementary material for: Development of emotional labor ability scale for kindergarten teachers
Source: PLoS One. 2025 Jun 23;20(6):e0325891. doi: 10.1371/journal.pone.0325891 (PMC12184924; doi:10.1371/journal.pone.0325891)
Supplement: S5 Table — α. (DOCX) [file pone.0325891.s008.docx]

| Table 5 Coefficient Cronbach’s α | | |
| --- | --- | --- |
| Factor | Cronbach’s α | Cronbach’s α |
| A emotional intelligence | 0.902 | 0.974 |
| B the ability of internalizing emotional labor rules | 0.904 |  |
| C the coordination ability in emotional labor | 0.953 |  |
| D the reflective ability after emotional labor | 0.943 |  |
| E the application ability to emotional labor strategies | 0.928 |  |
