# Supplementary material for: Development of emotional labor ability scale for kindergarten teachers
Source: PLoS One. 2025 Jun 23;20(6):e0325891. doi: 10.1371/journal.pone.0325891 (PMC12184924; doi:10.1371/journal.pone.0325891)
Supplement: S6 Table — (DOCX) [file pone.0325891.s009.docx]

| Table 6 Model AVE and CR Indexes Results | | |
| --- | --- | --- |
| Factor | AVE | CR |
| A emotional intelligence | 0.559 | 0.910 |
| B the ability of internalizing emotional labor rules | 0.604 | 0.900 |
| C the coordination ability in emotional labor | 0.767 | 0.952 |
| D the reflective ability after emotional labor | 0.708 | 0.951 |
| E the application ability to emotional labor strategies | 0.600 | 0.931 |
