# Supplementary material for: Development of emotional labor ability scale for kindergarten teachers
Source: PLoS One. 2025 Jun 23;20(6):e0325891. doi: 10.1371/journal.pone.0325891 (PMC12184924; doi:10.1371/journal.pone.0325891)
Supplement: S7 Table — (DOCX) [file pone.0325891.s010.docx]

| Table 7 Pearson Correlation and AVE Square Root Value  (Diagonal number is the square root of AVE) | | | | | |
| --- | --- | --- | --- | --- | --- |
| Factor | A | B | C | D | E |
| A emotional intelligence | 0.748 |  |  |  |  |
| B the ability of internalizing emotional labor rules | 0.685 | 0.801 |  |  |  |
| C the coordination ability in emotional labor | 0.679 | 0.730 | 0.876 |  |  |
| D the reflective ability after emotional labor | 0.710 | 0.778 | 0.824 | 0.841 |  |
| E the application ability to emotional labor strategies | 0.671 | 0.791 | 0.778 | 0.811 | 0.775 |
