# Supplementary material for: Development of emotional labor ability scale for kindergarten teachers
Source: PLoS One. 2025 Jun 23;20(6):e0325891. doi: 10.1371/journal.pone.0325891 (PMC12184924; doi:10.1371/journal.pone.0325891)
Supplement: S8 Table — (DOCX) [file pone.0325891.s011.docx]

| Table 8 Descriptive Statistics(N=818) | | | | | | |
| --- | --- | --- | --- | --- | --- | --- |
| Factor | range | minimum value | Maximum value | average value | standard deviation | variance |
| A emotional intelligence | 4.00 | 1.00 | 5.00 | 3.94 | 0.54 | 0.30 |
| B the ability of internalizing emotional labor rules | 4.00 | 1.00 | 5.00 | 4.05 | 0.59 | 0.35 |
| C the coordination ability in emotional labor | 4.00 | 1.00 | 5.00 | 4.00 | 0.60 | 0.37 |
| D the reflective ability after emotional labor | 4.00 | 1.00 | 5.00 | 3.99 | 0.57 | 0.33 |
| E the application ability to emotional labor strategies | 3.83 | 1.00 | 5.00 | 3.97 | 0.61 | 0.38 |
