# Supplementary material for: Development of emotional labor ability scale for kindergarten teachers
Source: PLoS One. 2025 Jun 23;20(6):e0325891. doi: 10.1371/journal.pone.0325891 (PMC12184924; doi:10.1371/journal.pone.0325891)
Supplement: Attachment — Submitted filename: PONE-D-24-16344 Reviewed.pdf [file pone.0325891.s012.pdf]

PLOS ONE

Development of Emotional Labor Ability Scale for Kindergarten Teachers

--Manuscript Draft--

**Manuscript Number:**	PONE-D-24-16344
**Article Type:**	Research Article
**Full Title:**	Development of Emotional Labor Ability Scale for Kindergarten Teachers
**Short Title:**	Development of Emotional Labor Ability Scale for Kindergarten Teachers
**Corresponding Author:**	Juan Hong<br>Xinjiang Normal University<br>Urumqi, CHINA
**Keywords:**	emotional labor; emotional labor ability; kindergarten teachers; scale development
**Abstract:**	Background : The lack of emotional labor ability of kindergarten teachers can lead to their inability to adapt to the high-tensity work in emotional labor, resulting in emotional exhaustion, job burnout, identity alienation, and low job satisfaction. At present, it has become the academic consensus to carry out systematic training of kindergarten teachers' Emotional labor ability. However, assessment instrument that meets the requirements of psychometrics for the emotional labor ability of kindergarten teachers has not been found.<br>Objective: The study aims to develop an emotional labor ability scale for kindergarten teachers and then to test the reliability and validity of the scale in the Chinese educational context.<br>methods: Firstly, based on the working mechanism model of emotional labor, the dimensions of emotional labor ability of kindergarten teachers were analyzed; Secondly, behavioral event interview(BEI) were conducted for 5 in-service kindergarten teachers, through which the primary dimensional elements and secondary competency elements of the emotional labor ability of kindergarten teachers were constructed by combining the real and specific emotional labor events; On this basis, two rounds of expert consultation involving five expert teachers were carried out, repeatedly revising the dimension elements and competency elements of the emotional labor ability of kindergarten teachers, which resulted in the Initial Questionnaire of the Emotional Labor Ability of Kindergarten Teachers; Finally, based on the data obtained in the questionnaire survey on 818 in-service kindergarten teachers, item analysis, exploratory factor analysis, confirmatory factor analysis, reliability analysis, convergence validity analysis, and discriminant validity analysis were carried out in the study.<br>Results : There are 30 items in the Emotional Labor Ability Scale for Kindergarten Teachers, including five dimensions, namely emotional intelligence, the ability of internalizing emotional labor rules, the coordination ability in emotional labor, the reflective ability after emotional labor, and the application ability to emotional labor strategies. The five dimensions explained 71.757% of the variance. As confirmatory factor analysis showed that, $\chi 2/df=2.774$, RMR=0.016, GFI=0.918, IFI=0.969, TLI=0.965, CFI=0.969, RMSEA=0.047, it is indicated that the five-factor model fits well. The internal consistency reliability coefficients for each factor are 0.902, 0.904, 0.953, 0.943, and 0.928, respectively.<br>Conclusion : The scale proves to be well-characterized for psychometrics and can be used as an effective tool to evaluate the emotional labor ability of kindergarten teachers .
**Order of Authors:**	Juan Hong
	Pan ri Yu
**Additional Information:**	
**Question**	**Response**
**Financial Disclosure**<br><br>Enter a financial disclosure statement that	Yes

Powered by Editorial Manager® and ProduXion Manager® from Aries Systems Corporation

describes the sources of funding for the work included in this submission. Review the submission guidelines for detailed requirements. View published research articles from *PLOS ONE* for specific examples.<br><br>This statement is required for submission and **will appear in the published article** if the submission is accepted. Please make sure it is accurate.<br><br>**Funded studies**<br>Enter a statement with the following details:<br>• Initials of the authors who received each award<br>• Grant numbers awarded to each author<br>• The full name of each funder<br>• URL of each funder website<br>• Did the sponsors or funders play any role in the study design, data collection and analysis, decision to publish, or preparation of the manuscript?<br><br>Did you receive funding for this work?	
Please add funding details.<br>as follow-up to "**Financial Disclosure**<br><br>Enter a financial disclosure statement that describes the sources of funding for the work included in this submission. Review the submission guidelines for detailed requirements. View published research articles from *PLOS ONE* for specific examples.<br><br>This statement is required for submission and **will appear in the published article** if the submission is accepted. Please make sure it is accurate.	This paper is a phased research result of the 2022 special project of the Xinjiang Uygur Autonomous Region Social Science Fund “Research on Education Assistance in Xinjiang form the Perspective of Nurturing Xinjiang with Culture” (Grant No: 22VZX008).

**Funded studies**<br>Enter a statement with the following details:<br>• Initials of the authors who received each award<br>• Grant numbers awarded to each author<br>• The full name of each funder<br>• URL of each funder website<br>• Did the sponsors or funders play any role in the study design, data collection and analysis, decision to publish, or preparation of the manuscript?<br><br>Did you receive funding for this work?"	
Please select the country of your main research funder (please select carefully as in some cases this is used in fee calculation).<br>as follow-up to "**Financial Disclosure**<br><br>Enter a financial disclosure statement that describes the sources of funding for the work included in this submission. Review the submission guidelines for detailed requirements. View published research articles from *PLOS ONE* for specific examples.<br><br>This statement is required for submission and **will appear in the published article** if the submission is accepted. Please make sure it is accurate.<br><br>**Funded studies**<br>Enter a statement with the following details:<br>• Initials of the authors who received each award<br>• Grant numbers awarded to each author<br>• The full name of each funder<br>• URL of each funder website<br>• Did the sponsors or funders play any role in the study design, data collection and analysis, decision to publish, or preparation of the manuscript?<br><br>Did you receive funding for this work?"	CHINA - CN
**Competing Interests**	The authors declare that this research was conducted in the absence of any

Use the instructions below to enter a competing interest statement for this submission. On behalf of all authors, disclose any competing interests that could be perceived to bias this work—acknowledging all financial support and any other relevant financial or non-financial competing interests.<br><br>This statement is required for submission and **will appear in the published article** if the submission is accepted. Please make sure it is accurate and that any funding sources listed in your Funding Information later in the submission form are also declared in your Financial Disclosure statement.<br><br>View published research articles from *PLOS ONE* for specific examples.<br><br>**NO authors have competing interests**<br><br>Enter: *The authors have declared that no competing interests exist.*<br><br>**Authors with competing interests**<br><br>Enter competing interest details beginning with this statement:<br><br>*I have read the journal's policy and the authors of this manuscript have the following competing interests: [insert competing interests here]*<br><br>* typeset	commercial or financial relationships that could be construed as potential conflicts of interest.
**Ethics Statement**<br><br>Enter an ethics statement for this submission. This statement is required if the study involved:<br><br>• Human participants<br>• Human specimens or tissue<br>• Vertebrate animals or cephalopods<br>• Vertebrate embryos or tissues	All procedures performed in studies involving human participants were in accordance with the ethical standards of the institutional and/or national research committee and with the 1964 Helsinki Declaration and its later amendments or comparable ethical standards. Informed consent was obtained from all individual participants included in the study. The study was approved by the Academic Ethics Review Committee of Huaihua University.

Powered by Editorial Manager® and ProduXion Manager® from Aries Systems Corporation

• Field research<br><br>Write "N/A" if the submission does not require an ethics statement.<br><br>General guidance is provided below. Consult the submission guidelines for detailed instructions. **Make sure that all information entered here is included in the Methods section of the manuscript.**	

**Format for specific study types**<br><br>**Human Subject Research (involving human participants and/or tissue)**<br>• Give the name of the institutional review board or ethics committee that approved the study<br>• Include the approval number and/or a statement indicating approval of this research<br>• Indicate the form of consent obtained (written/oral) or the reason that consent was not obtained (e.g. the data were analyzed anonymously)<br><br>**Animal Research (involving vertebrate animals, embryos or tissues)**<br>• Provide the name of the Institutional Animal Care and Use Committee (IACUC) or other relevant ethics board that reviewed the study protocol, and indicate whether they approved this research or granted a formal waiver of ethical approval<br>• Include an approval number if one was obtained<br>• If the study involved *non-human primates*, add *additional details* about animal welfare and steps taken to ameliorate suffering<br>• If anesthesia, euthanasia, or any kind of animal sacrifice is part of the study, include briefly which substances and/or methods were applied<br><br>**Field Research**<br><br>Include the following details if this study involves the collection of plant, animal, or other materials from a natural setting:<br>• Field permit number<br>• Name of the institution or relevant body that granted permission	
**Data Availability**<br><br>Authors are required to make all data underlying the findings described fully available, without restriction, and from the time of publication. PLOS allows rare exceptions to address legal and ethical concerns. See the PLOS Data Policy and FAQ for detailed information.	Yes - all data are fully available without restriction

A Data Availability Statement describing where the data can be found is required at submission. Your answers to this question constitute the Data Availability Statement and **will be published in the article**, if accepted.<br><br>**Important:** Stating 'data available on request from the author' is not sufficient. If your data are only available upon request, select 'No' for the first question and explain your exceptional situation in the text box.<br><br>Do the authors confirm that all data underlying the findings described in their manuscript are fully available without restriction?	
**Describe where the data may be found in full sentences. If you are copying our sample text, replace any instances of XXX with the appropriate details.**<br><br>• If the data are **held or will be held in a public repository**, include URLs, accession numbers or DOIs. If this information will only be available after acceptance, indicate this by ticking the box below. For example: *All XXX files are available from the XXX database (accession number(s) XXX, XXX.).*<br>• If the data are all contained **within the manuscript and/or Supporting Information files**, enter the following: *All relevant data are within the manuscript and its Supporting Information files.*<br>• If neither of these applies but you are able to provide **details of access elsewhere**, with or without limitations, please do so. For example:<br><br>*Data cannot be shared publicly because of [XXX]. Data are available from the XXX Institutional Data Access / Ethics Committee (contact via XXX) for researchers who meet the criteria for access to confidential data.*<br><br>*The data underlying the results presented in the study are available from (include the name of the third party*	All relevant data are within the manuscript and its Supporting Information files.

and contact information or URL).<br>• This text is appropriate if the data are owned by a third party and authors do not have permission to share the data.<br><br>* typeset	
Additional data availability information:	

Powered by Editorial Manager® and ProduXion Manager® from Aries Systems Corporation

手稿 Click here to access/download;Manuscript;Unblinded Manuscript.docx

Development of Emotional Labor Ability Scale for Kindergarten Teachers

Juan Hong[1,2], Ri-Yu Pan[3]

[1]College of Educational Science, Xinjiang Normal University, Urumqi, Xinjiang, China

[2]College of Educational Science, Huaihua University, Huaihua, Hunan, China

[3]School of Teacher Education, Anqing Normal University, Anqing, Anhui, China

Correspondence

Juan Hong

3066463476@qq.com

Abstract:

Background: The lack of emotional labor ability of kindergarten teachers can lead to their inability to adapt to the high-tensity work in emotional labor, resulting in emotional exhaustion, job burnout, identity alienation, and low job satisfaction. At present, it has become the academic consensus to carry out systematic training of kindergarten teachers' Emotional labor ability. However, assessment instrument that meets the requirements of psychometrics for the emotional labor ability of kindergarten teachers has not been found.

Objective: The study aims to develop an emotional labor ability scale for kindergarten teachers and then to test the reliability and validity of the scale in the Chinese educational context.

methods: Firstly, based on the working mechanism model of emotional labor, the dimensions of emotional labor ability of kindergarten teachers were analyzed; Secondly, behavioral event interview(BEI) were conducted for 5 in-service kindergarten teachers, through which the primary dimensional elements and secondary competency elements of

the emotional labor ability of kindergarten teachers were constructed by combining the real and specific emotional labor events; On this basis, two rounds of expert consultation involving five expert teachers were carried out, repeatedly revising the dimension elements and competency elements of the emotional labor ability of kindergarten teachers, which resulted in the Initial Questionnaire of the Emotional Labor Ability of Kindergarten Teachers; Finally, based on the data obtained in the questionnaire survey on 818 in-service kindergarten teachers, item analysis, exploratory factor analysis, confirmatory factor analysis, reliability analysis, convergence validity analysis, and discriminant validity analysis were carried out in the study.

Results: There are 30 items in the Emotional Labor Ability Scale for Kindergarten Teachers, including five dimensions, namely emotional intelligence, the ability of internalizing emotional labor rules, the coordination ability in emotional labor, the reflective ability after emotional labor, and the application ability to emotional labor strategies. The five dimensions explained 71.757% of the variance. As confirmatory factor analysis showed that, χ^2/df=2.774, RMR=0.016, GFI=0.918, IFI=0.969, TLI=0.965, CFI=0.969, RMSEA=0.047, it is indicated that the five-factor model fits well. The internal consistency reliability coefficients for each factor are 0.902, 0.904, 0.953, 0.943, and 0.928, respectively.

Conclusion: The scale proves to be well-characterized for psychometrics and can be used as an effective tool to evaluate the emotional labor ability of kindergarten teachers .

Keywords: emotional labor; emotional labor ability; kindergarten teachers; scale development

1. Introduction

Kindergarten teachers are high intensity emotional laborers. Existing research shows that emotional labor not only affects kindergarten teachers on their mental health (Nan-Sil & Suhyun, 2020), self-esteem (Kyung & Jang, 2021) and teaching effectiveness(Kwak & So-Hyun, 2022), but also has significant predictive effect on the social and emotional development of children (Xie et al., 2022) as well as the quality of teacher-child interaction (Shim, 2022 ; Min & Kim, 2020). However, in educational practice in China, the emotional labor ability of kindergarten teachers has not been given due consideration in the pre-vocational education, recruitment and post-service certification of kindergarten teachers. As a result, some newly-appointed kindergarten teachers have failed to adapt to the high intensity in emotional labor, which leads to their emotional exhaustion (No & Yeong-hee, 2021), job burnout (Peng et al., 2019), low job satisfaction (Eun, 2022), and reduced occupational well-being (Wei et al., 2021). The fundamental solution to the above problems, suggested by this study, is to improve the emotional labor ability of kindergarten teachers .At present, it has become the academic consensus to carry out systematic training of kindergarten teachers' Emotional labor ability (Cho & Sukyung, 2022).

In the early stages of research on emotional labor ability, existing studies have equated the concepts of "emotional intelligence" and "emotional labor ability" based on the interpersonal interaction characteristics of emotional labor (Mayer & Salovey, 1993). It is believed that emotional labor ability mainly includes emotional perception ability, emotional understanding ability, emotional management ability, and emotional application ability (Mayer et al., 2003 ; Mayer et al., 2004 ; Jennings & Greenberg, 2009).

In terms of the teacher group, some researchers have pointed out that teachers' emotional labor ability includes emotional perception ability, emotional understanding ability, emotional management ability, and emotional application ability Emotional regulation ability and emotional expression ability are three components (Rindermann, 2009). Jennings et al., (2013) proposed that emotional skills that teachers need to learn include emotional skills, mindfulness, and care and compassion. Based on the above research results, it is speculated that emotional intelligence is an important psychological resource for individuals to cope with emotional labor, but high emotional intelligence does not necessarily mean efficient emotional labor, because having ability is not equivalent to being able to successfully apply it. With the advancement of research on emotional labor ability, researchers have begun to focus on the specific work situation of workers to explore their emotional labor ability. They believe that emotional labor ability should be manifested as the ability of individuals to use various emotional labor strategies such as deep acting and natural expression based on specific educational situations (Yin et al., 2016 ; Wang et al., 2019 ; Chang & Taxer, 2020). As for the group of kindergarten teachers, the existing research has focused on the concept (Hong, 2018), importance (Zhang et al., 2022) and promotion strategy (Seom, 2022) of kindergarten teachers' Emotional labor ability by summarizing experience and elaborating views. This provides a useful reference for further exploration on the structural dimension of the emotional labor ability of kindergarten teachers.

[illegible]wever, there is still a lot of room for in-depth exploration. For example, the research results on the structural dimensions of the emotional labor ability of kindergarten teachers based on actual teaching scenes have been found rare in number,

and no existing research has been found on the tools for the evaluation of the emotional labor ability of kindergarten teachers. In view of this, based on the relevant theories on emotional labor and the practices of emotional labor of kindergarten teachers in China, this study explores the structural dimensions of the emotional labor ability of kindergarten teachers, then further clarifies the logical relationship among its internal factors, and finally develops an emotional labor ability scale for kindergarten teachers that meets the requirements of psychometrics so as to better evaluate and intervene the future training on the emotional labor ability of kindergarten teachers.

2. Methods

2.1 Research Design

This study follows the basic principles of scale development (Flora & Flake, 2017). Firstly, with the theoretical basis of the emotional labor ability of kindergarten teachers being excavated by literature research, the constituent elements of the emotional labor ability of kindergarten teachers are analyzed in this study; Secondly, adhering to the principle of combining theory with practice, behavioral event interviews(BEI) is conducted among in-service kindergarten teachers, and then, by combing the real and specific emotional labor events, this study constructs the primary dimension elements and secondary competency elements of the emotional labor ability of kindergarten teachers; On this basis, two rounds of expert consultation are carried out to revise the dimension elements and competency elements of the emotional labor ability of kindergarten teachers, which results in the Initial Questionnaire of the Emotional Labor Ability of Kindergarten Teachers; Finally, a questionnaire survey are conducted for kindergarten teachers to analyze the reliability and validity of the data obtained, so as to build a

scientific and reasonable structure model for the emotional labor ability of kindergarten teachers which results in the formal “Emotional Labor Ability Scale for Kindergarten Teachers”. It should be noted that this study was completed between October 25, 2023 and December 26, 2023. During the process of behavioral event interviews, expert consultations, and questionnaire surveys, all participants provided written informed consent forms.

2.2 Development and Administration of Scale

2.2.1 Theoretical Framework

According to the working mechanism model of emotional labor proposed by Grandey (Grandey & A, 2000), there are three stages in the working mechanism of emotional labor: situational cues, emotional regulation and long-term results (see Figure 1). In the stage of situational cues, individuals should start by using emotional intelligence to understand the interaction expectations of their work objects and the nature of emotional events, and then they need to have a correct understanding and deep recognition of the emotional labor rule to ensure that the following emotional expression behavior meets the organizational requirements. In the emotional regulation stage, individuals use emotional labor strategies to regulate their external emotional expression behavior and internal emotional feelings under the guidance of the emotional labor rules so that their subjective emotional experience can be transformed into the social action force with educational responsibilities. After emotional labor, individuals need to reflect on both the individual results and organizational results of their emotional labor, including on whether their emotional expression behavior conforms to professional behavior norms or whether it helps to achieve their purposes so as to help promote the

emotional labor level of themselves. It can be inferred that during emotional labor, individuals need to be equipped with emotional intelligence, the ability to internalize emotional labor rules, the ability to use emotional labor strategies, and the ability to reflect on their emotional labor.

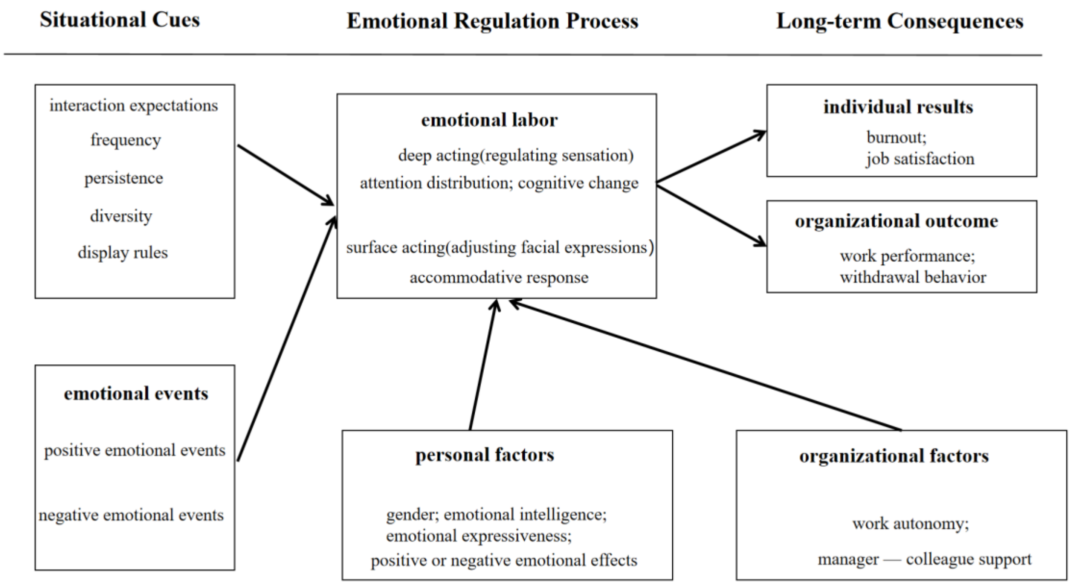

Figure 1 Working Mechanism of Emotional Labor

2.2.2 Source of Initial Items

To explore the special qualities of emotional labor ability of kindergarten teachers, this study conducted BEI for five in-service kindergarten teachers. The interviewees were from two different types of kindergartens in Hunan Province of China, namely public and private kindergartens, with a working experience of 3-7 years. Among them, there were 4 female teachers and 1 male teacher. The two interview questions were: What are the emotional labor events that you have done successfully in the past year? What do you think are the components of the emotional labor ability of kindergarten teachers? At the end of the interview, the interviewees were asked to provide their views on revising the

dimensions of the emotional labor ability of kindergarten teachers extracted from previous literature research.

Based on their own experience, one of the respondents pointed out, "*I think the ability to lead a class or implement a course can be seen as a guarantee. Because if my teaching ability is relatively weak, an ongoing course will be easily interrupted when emotional events occur. At this time, the emotional state of my own will be affected as well, and this will make the handling of emotional events even harder*(20220913YCY)." Thus it reflects a fact that emotional labor, and teaching activities of kindergartens integrate with each other, which requires a certain coordination ability of kindergarten teachers in emotional labor. In addition, another interviewee emphasized that, "*The guidance of professional codes of conduct, which I believe is the most important, is needed when dealing with emotional events. Otherwise, kindergarten teachers will be likely to lose control when dealing with emotional events, some of which became child abuse events in the past*(20220925DJ)." This view fully explained why the ability of internalizing emotional labor rules plays such an important role as a value guidance among the dimensions of the emotional labor ability of kindergarten teachers. By analyzing and summarizing all interview contents, it is assumed that the emotional labor ability of kindergarten teachers includes five dimensional elements, namely emotional intelligence, the ability of internalizing emotional labor rules, the application ability to emotional labor strategies, the coordination ability in emotional labor, and the reflective ability after emotional labor.

2.2.3 Delphi Method

Through literature research and BEI analysis, the dimensional elements and competency elements of the emotional labor ability of kindergarten teachers were obtained. Then, two rounds of expert consultation were carried out. The selected expert teachers, closely connected with the management of front-line kindergarten teachers, have been engaged in the teaching of basic theories of preschool education and educational research methods, kindergarten teacher education and other relevant teaching work in colleges or universities for a long time.

The authority coefficient of the five experts is 0.81, indicating a high credibility of the consultation results. The recovery rate of the two rounds of expert consultation questionnaires was 100%, indicating a high level of enthusiasm. In the first round of expert consultation, the average importance of the primary and secondary dimensional elements was: 4.75 points for emotional intelligence, 4.5 points for the ability of internalizing emotional labor rules, 4.75 points for the application ability to emotional labor strategies, 4.75 points for the coordination ability in emotional labor, and 4.5 points for the reflective ability after emotional labor. The average importance of each dimensional element was over 4 points. According to the results of the first round of expert consultation, the dimensional elements and competency elements of the emotional labor ability of kindergarten teachers were modified, and a feedback of the first round was given to all experts in the second round of consultation, and the experts were asked to assign their importance again. In the second round of consultation, the average importance of each dimensional element was: 5 points for emotional intelligence, 4.8 points for the ability of internalizing emotional labor rules, 5 points for the application ability to emotional labor strategies, 5 points for the coordination ability in emotional

labor, and 5 points for the reflective ability after emotional labor. The average importance of the above five dimensional elements and their secondary competency elements exceeded 4.5 points, with a full score ratio of 80% and a coefficient of variation of less than 20%, indicating a high consistency of expert opinions. This showed that the design of each dimensional elements and their competency elements for the emotional labor ability of kindergarten teachers is comparatively scientific, and the consistency of experts is high. As a result, an initial model of the emotional labor ability of kindergarten teachers, consisting of 5 basic elements and 16 competency elements was built(see Figure 2), based on which the Initial Questionnaire of the Emotional Labor Ability of Kindergarten Teachers was compiled.

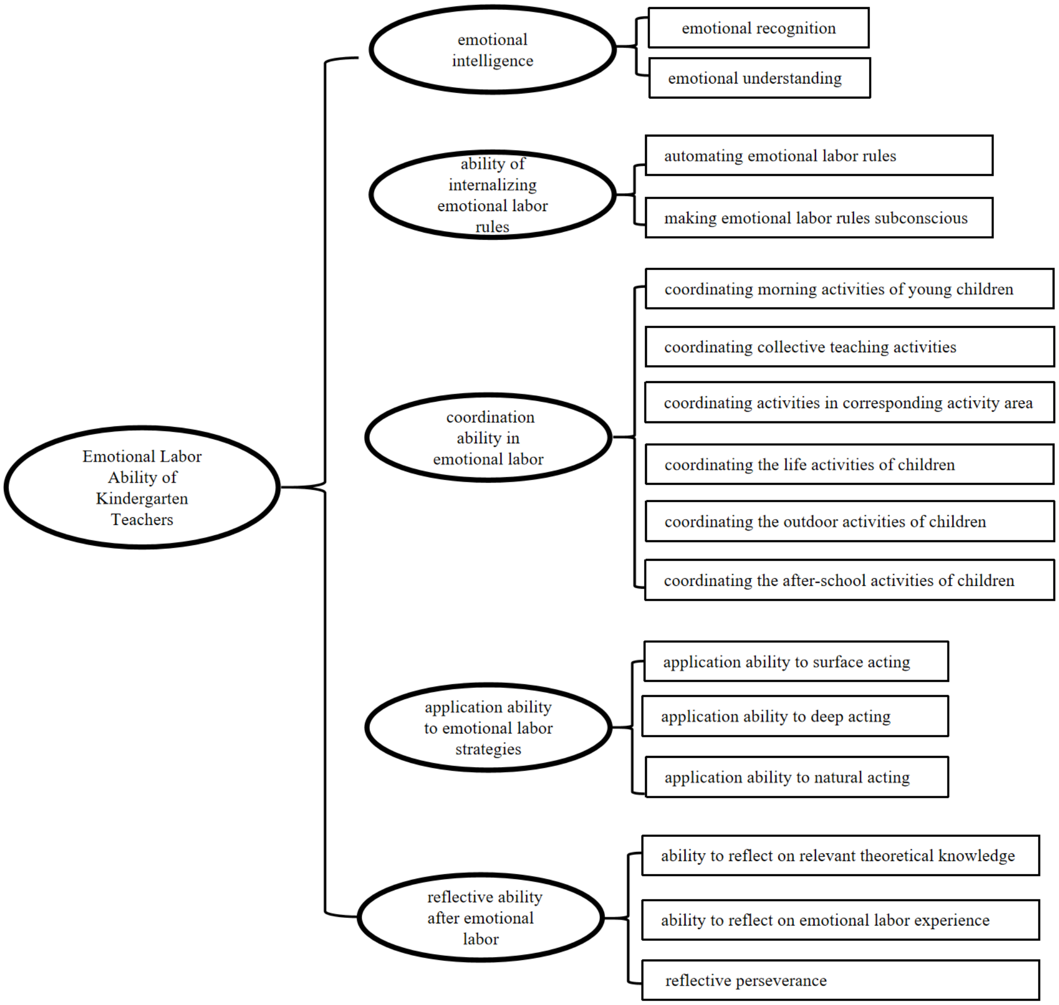

Figure 2 The Initial Model for the Emotional Labor Ability of Kindergarten Teachers

2.2.4 [illegible]stionnaire survey

The survey tool is the Initial Questionnaire of the Emotional Labor Ability of Kindergarten Teachers, which is based on the results of previous literature research, interviews and expert consultations. The questionnaire consists of five dimensional elements and 37 items, and each of the question item adopts the Likert five point scoring method. On the questionnaire network platform of WJX.CN, a convenience sampling was conducted to distribute questionnaires to in-service kindergarten teachers in Hunan

Province, with a total of [illegible] pieces of valid data collected. Then, the collected valid data was randomly split into two. The first 409 pieces of data were used for exploratory factor analysis, and a confirmatory factor analysis was conducted on the other 409 pieces.

3.[illegible]ults

3.1 Item Analysis

The total score of each subject on the scale was calculated and then ranked in ascending order. The top 27% were the low-score groups while the bottom 27% were the high-score groups. Conduct independent sample t-tests on the scores of the high and low groups on the 37 items, and then calculate the Pearson correlation between each item and the total score. The results, as can be seen from Table 1, showed that the values of t range from -16.07 to -7.07, and the p-values are all less than 0.001. Correlation analysis showed that the correlation coefficient between each item and the total score is between 0.57 and 0.81, all exceeding 0.4, and the p-value is less than 0.001. The internal consistency test showed that the Cronbach's alpha coefficient of the whole scale is 0.98. Additionally, the Cronbach's alpha coefficient when deleting a certain item was calculated,too. And the results showed that deleting any item would result in a decrease in reliability. Therefore, the item analysis of this study did not delete any item.

Table1 Correlation Coefficients Between Items and Total Score (N=409)

Item	Low Group (N=111, M±SD)	High Group (N=111, M±SD)	C.R. Value	Correlation Coefficients (N=409)	α if Deleted
A1	3.72±0.80	4.39±0.59	-7.07***	0.63***	0.97
A2	3.63±0.79	4.49±0.59	-9.20***	0.68***	0.97
A3	3.54±0.84	4.39±0.58	-8.77***	0.71***	0.97

A4	3.45±0.85	4.40±0.61	-9.54***	0.67***	0.97
A5	3.65±0.81	4.48±0.59	-8.77***	0.69***	0.97
A6	3.51±0.81	4.41±0.61	-9.29***	0.66***	0.97
A7	3.32±0.81	4.38±0.60	-10.98***	0.70***	0.97
A8	3.45±0.75	4.39±0.61	-10.26***	0.66***	0.97
B1	3.44±0.83	4.70±0.50	-13.76***	0.72***	0.97
B2	3.23±0.84	4.44±0.64	-12.01***	0.63***	0.97
B3	3.67±0.89	4.71±0.46	-11.04***	0.74***	0.97
B4	3.58±0.73	4.62±0.51	-12.37***	0.75***	0.97
B5	3.50±0.87	4.70±0.46	-12.80***	0.76***	0.97
B6	3.34±0.99	4.63±0.50	-12.26***	0.68***	0.97
E1	3.19±0.79	4.46±0.67	-12.89***	0.66***	0.97
E2	3.29±0.82	4.42±0.73	-10.84***	0.63***	0.97
E3	3.26±0.83	4.42±0.70	-11.33***	0.65***	0.97
E4	3.44±0.88	4.70±0.46	-13.38***	0.72***	0.97
E5	3.38±0.89	4.76±0.47	-14.49***	0.77***	0.97
E6	3.32±0.83	4.77±0.45	-16.07***	0.79***	0.97
E7	3.58±0.80	4.76±0.47	-13.34***	0.81***	0.97
E8	3.10±0.92	4.40±0.97	-10.23***	0.57***	0.97
E9	3.39±0.86	4.68±0.56	-13.29***	0.74***	0.97
C1	3.51±0.84	4.59±0.53	-11.36***	0.79***	0.97
C2	3.53±0.75	4.58±0.61	-11.39***	0.75***	0.97
C3	3.61±0.77	4.67±0.51	-12.08***	0.79***	0.97
C4	3.59±0.73	4.71±0.48	-13.60***	0.76***	0.97
C5	3.49±0.85	4.72±0.45	-13.50***	0.81***	0.97
C6	3.59±0.83	4.68±0.49	-12.09***	0.80***	0.97

D1	3.53±0.85	4.68±0.51	-12.17***	0.78***	0.97
D2	3.58±0.83	4.68±0.56	-11.73***	0.78***	0.97
D3	3.50±0.80	4.60±0.51	-12.35***	0.77***	0.97
D4	3.43±0.77	4.61±0.51	-13.48***	0.78***	0.97
D5	3.50±0.74	4.59±0.51	-12.80***	0.80***	0.97
D6	3.43±0.86	4.59±0.51	-12.24***	0.78***	0.97
D7	3.38±0.79	4.56±0.57	-12.82***	0.77***	0.97
D8	3.30±0.82	4.56±0.53	-13.63***	0.80***	0.97
Notes: *p<0.05,**p<0.01, ***p<0.001.					
Abbreviations: C.R. Value, critical ratio value.					

3.2 Exploratory Factor Analysis

The Initial Questionnaire of the Emotional Labor Ability of Kindergarten Teachers was tested by Kaiser-Meyer-Olkin (KMO) and Bartlett's test of sphericity. The results showed that the KMO coefficient was 0.966, the value of χ^2 was 13568.248, the degree of freedom was 666, and $p<0.001$, indicating that it is advisable to conduct exploratory factor analysis.

Using principal component analysis and maximum variance method to extract common factors, and then deleting items with factor load value less than 0.5, commonality less than 0.4, multiple loads with similar load values, or improper factor classification, a total of 4 items were deleted, with 33 items remaining. Finally, five common factors were obtained, with a cumulative total variance explained of 71.757%. Among them, delete the four items "E1, E2, E3, B2", and the resulting item load values and commonality are shown in Table 2.

Table 2 Exploratory Factor Analysis Results of the Questionnaire of the Emotional Labor Ability of Kindergarten Teachers

Item	Factor Loading					Commonality
	1	2	3	4	5	
A2	.737					.703
A1	.731					.664
A5	.668					.684
A4	.640					.619
A3	.626					.629
A6	.616					.625
A8	.605					.621
A7	.581					.637
C3		.779				.845
C2		.756				.779
C1		.744				.814
C4		.725				.765
C6		.707				.809
C5		.654				.771
D6			.726			.788
D7			.704			.759
D8			.661			.757
D4			.652			.738
D5			.636			.746
D3			.602			.694
D2			.596			.713
D1			.560			.717
E8				.722		.620
E6				.679		.781
E5				.666		.751
E9				.647		.713
E4				.632		.663
E7				.607		.762
B6					.736	.715
B5					.705	.780
B3					.658	.718
B4					.605	.680
B1					.602	.619
Eigenvalue	18.337	1.592	1.464	1.228	1.058	Total

Total variance explained	55.567%	4.825%	4.437%	3.722%	3.206%	71.757%

3.3 Confirmatory Factor Analysis

Confirmatory factor analysis was conducted using the software of Amos 21.0. According to the modification indices and factor loads, A8, D1, and D2 were deleted and correlations for residuals between certain items were established based on theoretical analysis.

To establish an optimal model, multiple validations were conducted on the model(see Table 3) and it is found that the χ^2/df, GFI, IFI, TLI, CFI, and RMSEA in one factor model did not reach the recommended values, while all indicators of the five-factor model met the recommendation requirements. This indicated that the five-factor model fits well and is in line with the actual situation. The final model is shown in Figure 3.

Table 3 Fitting Indicator of the Model of the Emotional Labor Ability of Teachers

Fitting indicator	χ^2/df	RMR	GFI	IFI	TLI	CFI	RMSEA
Recommended value	<3	<0.05	>0.90	>0.90	>0.90	>0.90	<0.08
Fitted value of one-factor model	9.099	0.027	0.720	0.853	0.839	0.853	0.100
Fitted value of five-factor model	2.774	0.016	0.918	0.969	0.965	0.969	0.047

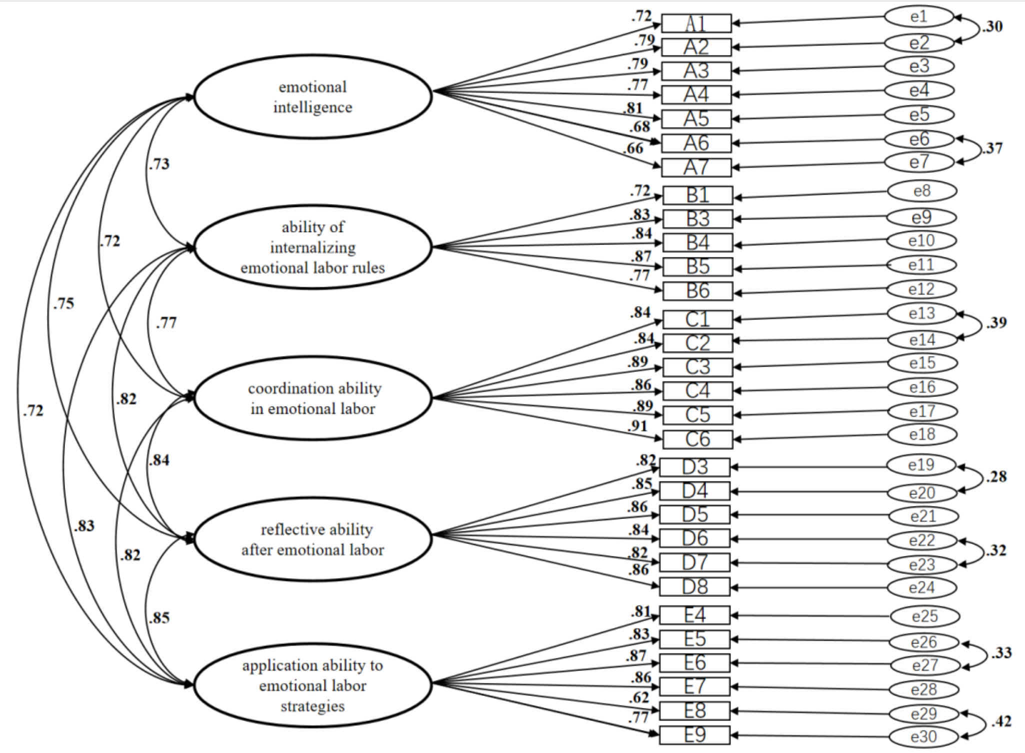

Figure 3 The Final Model of the Emotional Labor Ability of Kindergarten Teachers

3.4 Reliability and Validity Testing

As can be seen from Table 4 below, the reliability analysis showed that the Cronbach's α of the Emotional Labor Ability Scale for Kindergarten Teachers is 0.974, indicating a good reliability of the scale. In addition, for each factor, the coefficients Cronbach's α are all above 0.902, indicating a good reliability of each factor.

Table 4 Coefficient Cronbach's α

Factor	Cronbach's α	Cronbach's α
A emotional intelligence	0.902	0.974
B the ability of internalizing emotional labor rules	0.904	

C the coordination ability in emotional labor	0.953	
D the reflective ability after emotional labor	0.943	
E the application ability to emotional labor strategies	0.928	

Confirmatory factor analysis (CFA) was conducted for five factors and thirty items. As shown in Table 5, the AVE values of the three factors were all greater than 0.5, and the CR values were all higher than 0.7, which means that the scale had good convergence validity.

Table 5 Model AVE and CR Indexes Results

Factor	AVE	CR
A emotional intelligence	0.559	0.910
B the ability of internalizing emotional labor rules	0.604	0.900
C the coordination ability in emotional labor	0.767	0.952
D the reflective ability after emotional labor	0.708	0.951
E the application ability to emotional labor strategies	0.600	0.931

According to the analysis of discriminant validity (Table 6), the square root of AVE square root values for the factors were 0.748, 0.801, 0.876, 0.841 and 0.775, which were greater than the maximum value of absolute correlation coefficient between factors, meaning that the scale had good discriminant validity.

Table 6 Pearson Correlation and AVE Square Root Value
(Diagonal number is the square root of AVE)

Factor	A	B	C	D	E
A emotional intelligence	0.748				
B the ability of internalizing emotional	0.685	0.801			

labor rules					
C the coordination ability in emotional labor	0.679	0.730	0.876		
D the reflective ability after emotional labor	0.710	0.778	0.824	0.841	
E the application ability to emotional labor strategies	0.671	0.791	0.778	0.811	0.775

4. [illegible]ussion

This study aims to develop the Emotional Labor Ability Scale for Kindergarten Teachers in order to provide an effective tool for evaluating the emotional labor ability of kindergarten teachers. Therefore, based on the working mechanism model of emotional labor, this study conducted behavioral event interviews with front-line kindergarten teachers, followed by two rounds of expert consultation. As a result, the dimensional elements and competency elements of the emotional labor ability of kindergarten teachers were built and the Initial Questionnaire of the Emotional Labor Ability of Kindergarten Teachers was complied. After that, questionnaire surveys were conducted among kindergarten teachers, and then item analysis, exploratory factor analysis, confirmatory factor analysis, reliability analysis, convergence validity analysis, and discriminant validity analysis on the valid data obtained were conducted, which proved that the Emotional Labor Ability Scale for Kindergarten Teachers is highly reliable and valid. T[illegible] scale has 30 items and 5 dimensions, namely emotional intelligence, the ability of internalizing emotional labor rules, the coordination ability in emotional labor, the application ability to emotional labor strategies, and the reflective ability after emotional labor.

4.1 Emotional intelligence: permeating all stages of the emotional labor of kindergarten teachers

Emotional intelligence refers to the ability of kindergarten teachers to recognize and understand the emotional state of others in interpersonal interaction, and to use this information to guide the whole process of emotional labor. It includes two sub-dimensions, namely the ability of emotional recognition and the ability of emotional understanding, and a total of 8 items. Young children have variable emotions but weaker ability in language expression and they have not yet, been able to accurately express their emotional feelings and interactive demands. Therefore, in the stage of situational cues, kindergarten teachers need to use their emotional intelligence to identify and understand the emotional information conveyed by children, which has a significant impact on the emotional labor level of kindergarten teachers (Byeon & Yim, 2021). In the emotional regulation stage, kindergarten teachers with high emotional intelligence can use emotional regulation strategies more flexibly (Double et al., 2022). After emotional labor, emotional intelligence can also alleviate the emotional distress (Cheng et al., 2020) and job burnout (Kyung-chul et al., 2021) caused by kindergarten teachers during their work process. Based on this, emotional intelligence is the psychological cornerstone for kindergarten teachers to carry out emotional labor, and it plays a fundamental, guiding and overall role in all stages of emotional labor of kindergarten teachers. The higher the score in this dimension, the more acutely kindergarten teachers can recognize the emotional information transmitted in the process of emotional labor, and thus correctly understand the meaning of emotional expression and respond in a timely manner.

4.2 The ability of internalizing emotional labor rules: a value guidance for emotional labor

The ability of internalizing emotional labor rules refers to the ability of kindergarten teachers to deeply identify with and consciously practice the code of conduct of emotional labor for the normal operation of emotional labor, including the subconsciousness of emotional labor rules and the automation of emotional labor rules, with a total of 5 items. Research has shown that the emotional labor rule is the bumper for kindergarten teachers in the process of Emotional labor (Quan, 2009). When kindergarten teachers identify with the rules of emotional labor, they can avoid emotional exhaustion in the process of Emotional labor. Therefore, kindergarten teachers need to consciously follow the corresponding rules in emotional labor. Only when kindergarten teachers internalize the rules of emotional labor in their minds and externalize them in their actions, can they follow their own inclinations without violating the rules in emotional labor. More importantly, the extend to which kindergarten teachers identify with emotional labor rules will directly affect their choices of emotional labor strategies (Lee & Suh, 2015; Shin et al., 2020). Therefore, it can be concluded that the ability of internalizing emotional labor rules is the value guidance for kindergarten teachers to carry out Emotional labor, and determines and maintains that kindergarten teachers' Emotional labor conforms to the standard. The higher the score in this dimension, the more kindergarten teachers identify with the requirements of the organization for emotional labor, and the more they can protect the interests of the organization as well as their professional image in emotional labor.

4.3 The coordination ability in emotional labor: a guarantee for smooth progress of emotional labor

The coordination ability in emotional labor refers to the ability of kindergarten teachers to deal with emotional events in complex educational situations while ensure the smooth progress of all aspects of the kindergarten's daily activities. It mainly includes six sub-dimensions, namely the ability to coordinate collective teaching activities of the kindergarten, the ability to coordinate the morning activities of children, the ability to coordinate the life activities of children, the ability to coordinate the activities in corresponding activity area, and the ability to coordinate the outdoor or after-school activities of children, totaling 6 items.The emotional labor of kindergarten teachers permeates in all aspects of the kindergarten's daily activities, and they are integrated and inseparable. Existing research also suggests that different emotions can become professionalized based on the job content of the laborer themselves (Hochschild, 1975). As far as preschool education is concerned, emotional labor is indeed, in a professional development where it co-exists and coordinates with kindergarten teaching activities. Bringing the coordination ability in emotional labor into the concept of the emotional labor ability of kindergarten teachers also responds to the view of previous studies, that is, emotional labor should no longer be regarded as a means of exploitation (Wainwright et al., 2010) but as an indispensable part of professional practical activities in preschool education (Osgood, 2010). The higher the score in this dimension, the more control kindergarten teachers have in emotional ability, and the more like they can strike a perfect balance between high-intensity emotional labor and professional teaching activities.

4.4 The application ability to emotional labor strategies: the key to effective operatio n of emotional labor

The application ability to emotional labor strategies refers to the ability of kindergarten teachers to use certain emotional labor strategies according to the needs of educational situations in the process of emotional labor, including two sub-dimensions, namely the ability to use deep performance strategy and the ability to use natural performance strategy, with a total of 6 items. It is worth noting that emotional labor strategies include the strategies of surface acting, deep acting and natural expression (Diefendorff & Richard, 2003). However, in the process of exploratory factor analysis, the E1, E2, and E3 items under the dimension of “the application ability to surface acting strategy” were excluded due to the occurrence of cross loading. The reason may be that the surface acting strategy belongs to a remedial process of emotional labor (Grandey & Alicia, 2000), that is, When there is a certain gap between the kindergarten teachers' inner emotional feelings and the Emotional labor rules stipulated by the organization, the kindergarten teachers adopt surface acting strategy to change the external emotional expression to meet the organizational requirements, but not fully agree with the Emotional labor rules in the heart, resulting in negative consequences such as emotional imbalance, emotional exhaustion and job burnout (Hülsheger et al., 2010). Therefore, the use of surface acting strategy cannot be regarded as a symbol of competence in emotional labor. In general, the higher the score of this dimension, the more frequently kindergarten teachers adopt deep acting and natural expression strategies in the process of emotional labor, and the more appropriate emotions they can express in complex and changing educational situations to meet organizational requirements.

4.5 The reflective ability after emotional labor: the power source for the circulation of emotional labor

The essence of reflective ability after emotional labor is that kindergarten teachers actively review their own emotional labor process to find their own problems in emotional labor and to find effective ways to solve problems, including three sub-dimensions, namely reflective perseverance, the ability to reflect on relevant theoretical knowledge and the ability to reflect on emotional labor experience, with a total of 8 items. The reflective ability after emotional labor is an important symbol of the subjectivity of kindergarten teachers in emotional labor. It is also a high-level cognitive activity for kindergarten teachers to critically review the process and results of emotional labor. Moreover, reflective ability is an important component of professional growth (Beauchamp & Catherine, 2015) as well as an inevitable requirement for the development of teachers as a reflective educator (Lasley, 1992). Therefore, improving reflective ability after emotional labor is the internal need of kindergarten teachers in their professional development. The higher the score in this dimension, the more actively kindergarten teachers can participate in the process of emotional labor, the more independent thinking and rational analysis can be carried out in the process of emotional labor so that their emotional labor level can be constantly improved.

This study has certain theoretical significance. First, the five-factor structure of the emotional labor ability of kindergarten teachers constructed in this study is basically consistent with the working mechanism model of emotional labor proposed by Grundi, which helps to demonstrate the explanatory power of the working mechanism model of emotional labor in the field of education. Secondly, previous studies mainly focused on

one aspect of emotional labor ability, such as emotional intelligence, application ability to emotional labor strategy, etc. Yet this study conducted a systematic and comprehensive analysis on the constituent elements of the emotional labor ability of kindergarten teachers, and proposed a five-factor model of the emotional labor ability of kindergarten teachers, which can enrich the connotation of the emotional labor ability of kindergarten teachers. Finally, most of the existing studies use a single speculative research method to describe the components of the emotional labor ability of kindergarten teachers. Thus it remains unknown whether the deductive reasoning by personal perceptual experience can withstand data verification, which leads to a relative lack of feasibility and applicability of research conclusions. This study used mixed research methods to verify and analyze the internal structure of the emotional labor ability of kindergarten teachers, and developed the Emotional Labor Ability Scale for Kindergarten Teachers that meets the requirements of psychological measurement, which has certain practicality and can be provided as an effective tool for the development of quantitative research in this field in the future.

This study has certain practical significance, too. The Emotional Labor Ability Scale for Kindergarten Teachers developed in this study can be used by kindergarten managers for the evaluation of the emotional labor ability of applicants, so as to change the situation where kindergarten teacher recruitment only focuses on the teaching ability of kindergarten teachers and ignores their emotional emotional ability. In doing so, job seekers who do not match the kindergarten-teacher career can be eliminated from the start to maximize the personnel and job matching of kindergarten teachers so that the stability of the kindergarten teacher team can be effectively promoted. Additionally, the

scale is also an evaluation tool for the effectiveness of training on the emotional labor ability of kindergarten teachers. The measurement can be conducted before and after the training to judge whether it is effective and to ensure the most scientific and effective training methods are adopted so that the post-service training resources can be efficiently coordinated.

5. Limitations and Future Research

Although the reliability and validity test results of the scale shows that the Emotional Labor Ability Scale for Kindergarten Teachers developed in this study can be used as an effective tool to evaluate the emotional labor ability of kindergarten teachers, there are still some limitations and shortcomings.

First, the emotional labor ability of kindergarten teachers is a complex concept involving psychology, pedagogy, sociology and other multi-disciplinary fields. This study divides it into five dimensions: emotional intelligence, the ability of internalizing emotional labor rules, the coordination ability in emotional labor, the application ability to emotional labor strategies, and the reflective ability after emotional labor, which may not cover all the connotations of the concept. Therefore, whether the five-dimension structure proposed in this study is the optimal structure for the emotional labor ability of kindergarten teachers remains to be further verified by future research. Second, this study only involves questionnaire surveys and reliability and validity tests on the kindergarten teachers in Hunan Province of China, so the sample size may not be able to represent the kindergarten teachers in different urban or rural areas and regions across China. Thus future research needs to further expand the survey subjects. Third, in order to protect the privacy of the respondents, the questionnaire survey was conducted anonymously,

making it impossible to track the respondents. Therefore, we did not analyze the retest reliability of the scale, which may affect the stability of it. Fourth, the Emotional Labor Ability Scale for Kindergarten Teachers developed in this study is more suitable for the kindergarten teachers in the Chinese educational context, and its relevance in a cross-cultural context needs further testing. Therefore, more research is needed to determine whether the scale has certain cross-cultural invariance.

6. [illegible]clusion

The Emotional Labor Ability Scale for Kindergarten Teachers developed in this study consists of five dimensional elements, namely emotional intelligence, the ability of internalizing emotional labor rules, the coordination ability in emotional labor, the application ability to emotional labor strategies, and the reflective ability after emotional labor, with 15 competency elements and a total of 30 items. The results of item analysis, exploratory factor analysis, confirmatory factor analysis, reliability analysis, convergence validity analysis, and discriminant validity analysis showed that the scale is well-characterized for psychometrics and can be used as an effective tool to evaluate the emotional labor ability of kindergarten teachers.

Data availability

The data supporting the conclusions of this article are available upon request to correspondence author.

Author Contributions

Juan Hong designed this study, distributed and recycled the questionnaire, wrote the first draft, and repeatedly revised and improved the draft. Ri-Yu Pan conducted statistical analysis of the questionnaire data.

Funding

This paper is a phased research result of the 2022 special project of the Xinjiang Uygur Autonomous Region Social Science Fund "Research on Education Assistance in Xinjiang form the Perspective of Nurturing Xinjiang with Culture" (Grant No: 22VZX008).

Conflict of interest

The authors declare that this research was conducted in the absence of any commerc ial or financial relationships that could be construed as potential conflicts of interest.

Ethical approval

All procedures performed in studies involving human participants were in accordance with the ethical standards of the institutional and/or national research committee and with the 1964 Helsinki Declaration and its later amendments or comparable ethical standards. Informed consent was obtained from all individual participants included in the study. The study was approved by the Academic Ethics Review Committee of Huaihua University.

Informed consent

All participants provided their online informed consent prior to participation in this s tudy.

References

[1]Byeon, Hyeonsu., Yim, Myung-Seong.. (2021). Mediating Effect of Resilience on the Relationship between Emotional Intelligence and Emotional Labor. *Logos Management Review, 19*(2), 97-114. https://doi.org/10.22724/LMR.2021.19.2.97.

[2]Beauchamp, & Catherine. (2015). Reflection in teacher education: issues emerging fro

m a review of current literature. *Reflective Practice, 16*(1), 123-141. https://doi.org/10.1080/14623943.2014.982525.

[3]Chang, M. L., & Taxer, J.. (2020). Teacher emotion regulation strategies in response to classroom misbehavior. *Teachers and Teaching*(1), 1-17. https://doi.org/10.1080/13540602.2020.1740198.

[4]Cho, Eunjung., SUKYUNG, SHIN.. (2022). Mediating Effect of Autonomy on the Relationship Between Self-leadership and Emotional Labor of Early Childhood Teachers. *The Journal of Humanities and Social science, 13*(6), 827-842. https://doi.org/10.22143/hss21.13.6.56.

[5]Cheng, X., Ma, Y., Li, J., Cai, Y., & Zhang, J.. (2020). Mindfulness and psychological distress in kindergarten teachers: the mediating role of emotional intelligence. *International Journal of Environmental Research and Public Health, 17*(21), 8212. https://doi.org/10.3390/ijerph17218212.

[6]Diefendorff, J. M., & Richard, E. (2003). Antecedents and Consequences of Emotional Display Rule Perceptions. *Journal of Applied Psychology, 88*(02), 284–294.https://doi.org/10.1037/0021-9010.88.2.284.

[7]Double, Kit S., Pinkus, Rebecca T., MacCann, Carolyn.. (2022). Emotionally intelligent people show more flexible regulation of emotions in daily life. *Emotion*, *22*(2),397-402. https://doi.org/10.1037/emo0001069.

[8]EUN, YOU JI. (2022). Analyze Factors to Influence the Turnover Intention of Childcare Teachers. *The Journal of the Korea Contents Association, 22*(11), 660-672. https://doi.org/10.5392/JKCA.2022.22.11.660.

[9]Flora, D. B., & Flake, J. K.. (2017). The purpose and practice of exploratory and confi

rmatory factor analysis in psychological research: decisions for scale development and validation. *Canadian Journal of Behavioural Science*, *49*(2), 78-88. https://doi.org/10.1037/cbs0000069.

[10]Grandey, & Alicia, A.. (2000). Emotion regulation in the workplace: a new way to conceptualize emotional labor. *J Occup Health Psychol, 5*(1), 95-110. https://doi.org/10.1037/1076-8998.5.1.95.

[11]Hochschild, A. R. (1975). The Sociology of Feelings and Emotion:Selected Possibilities. *Sociological Inquiry, 45*(2-3), 289-290.

[12]Hong, sung-hee.. (2018). The Study on the Teacher's Emotional Labor in Early Childhood Education Institution. *DONGAINMUNHAK*, 45, 473-494. https://doi.org/10.52639/JEAH.2018.12.45.473.

[13]Hülsheger, Ute R., Lang, J. W. B., & Maier, Günter W. (2010). Emotional labor, strain, and performance: testing reciprocal relationships in a longitudinal panel study. *Journal of Occupational Health Psychology, 15*(4), 505-521. https://doi.org/10.1037/a0021003.

[14]Jennings, P. A., & Greenberg, M. T. (2009). The prosocial classroom: Teacher social and emotional competence in relation to student and classroom outcomes. *Review of Educational Research*, *79*(1), 491e525. https://doi.org/10.3102/0034654308325693.

[15]Jennings, P. A., Frank, J. L., Snowberg, K. E., Coccia, M. A., & Greenberg, M. T. (2013). Improving classroom learning environments by Cultivating Awareness and Resilience in Education (CARE): Results of a randomized controlled trial. *School Psychology Quarterly, 28*(4), 374e390. https://doi.org/10.1037/spq0000035.

[16]Kyung, Moon Young., Jang, Hye Ju.. (2021). Childcare Teacher's Emotional Labor Clusters: The Differences of Inter-Personal Stress and Self-Esteem by Clusters. *The Socie*

ty of Convergence Knowledge Transactions, 9(1), 75-83. https://doi.org/10.22716/sckt.2021.9.1.008.

[17]Kwak, Ester., So-Hyun, Jang.. (2022). The Effect of Early Childhood Teacher’s Emotional Labor on Child-teacher Interaction: Serial Multiple Mediation of Teaching Efficacy and Burnout. *Journal of Children’s Literature and Education, 23*(3), 297-312. https://doi.org/10.22154/jcle.23.3.13.

[18]Kyung-chul, Kim., Xinyu, Zhang., Chunjing, Cai.. (2021). The Effect of Interpersonal Relationships in the Workplace of Kindergarten Teachers in China on Job Burnout: Focusing on the Emotional Intelligence Modulating Effect. *International Journal of Early Childhood Education, 27*(2), 89-115.

[19]Lasley, T. J.. (1992). Promoting teacher reflection. *Journal of Staff Development,*13, 24-29.

[20]Lee, Y. J., & Suh, Y. S.. (2015). Emotional display rules and emotional labor strategy of childcare teachers. *Korean Journal of Childcare and Education, 11*(5), 19-37. https://doi.org/10.14698/jkcce.2015.11.5.019.

[21]Min, Y. P. , & Kim, K. R.. (2020). The effect of early childhood teachers' emotional labor on teacher-child interaction. *Korea Open Association for Early Childhood Education, 25*(2), 299-324.https://doi.org/10.20437/KOAECE25-2-13.

[22]Mayer, J. D., & Salovey, P.. (1993). The intelligence of emotional intelligence. *Imagination Cognition and Personality, 17*(3), 433-442. https://doi.org/10.1016/0160-2896(93)90010-3.

[23]Mayer, J. D., Salovey, P., Caruso, D. R., & Sitarenios, G.. (2003). Measuring emotional intelligence with the msceit v2.0. *Emotion, 3*(1), 97-105. https://doi.org/10.1037/1528

-3542.3.1.97.

[24]Mayer, J. D., Salovey, P., & Caruso, D. R. (2004). Emotional intelligence: Theory, findings, and implications. *Psychological Inquiry, 15*(3), 197e215. https://doi.org/10.1207/s15327965pli1503_02.

[25]No, Soon-Ok., Yeong-hee, Kim.. (2021) Effects of Preschool Teacher's Emotional Labor on Burnout, Job Satisfaction, and Turnover Intention. *Life Science Research Forum, 25*(1), 69-81. https://doi.org/10.36357/johe.2021.25.1.69.

[26]Nan-Sil, Kim., Suhyun, Kwon.. (2020). The Effects of Childcare Teachers' Emotional Labor on Psychological Well-being. *The Journal of Humanities and Social science, 11*(5), 1659-1674. https://doi.org/10.22143/hss21.11.5.118.

[27]Osgood, J.. (2010). Reconstructing professionalism in ecec: the case for the 'critically reflective emotional professional'. *Early Years, 30*(2), 119-133. https://doi.org/10.1080/09575146.2010.490905.

[28]Peng, J., He, Y., Deng, J., Zheng, L., Chang, Y., & Liu, X.. (2019). Emotional labor strategies and job burnout in preschool teachers: psychological capital as a mediator and moderator. *Journal of neurosurgical sciences*(3), 63. https://doi.org/10.3233/WOR-192939.

[29]Quan H. Z.. (2009). Childcare Center Teachers' Emotional Labor and Emotional Exhaustion: The Moderating Effects of Emotional Display Rules.*Early Childhood Education Research & Review*,13(6),255-276.

[30]Rindermann, H. (2009). Ekf: Emotionale-Kompetenz-Fragebogen. Hogrefe.

[31]Shin, Dongju., Myoung, Um. So.. (2020). A Study on the Relations among Emotional Labor Types, Perception of Emotional Display Rules and Emotion Regulation Strategies of Childcare Center Teachers. *Korean Journal of Child Education and Care, 20*(3),25-40

. https://doi.org/10.21213/kjcec.2020.20.3.25.
[32]Seom, Han San.. (2022). An Analysis on Kindergarten Teachers' Emotional Labor. *The Journal of EducationKyungin University of Education Educational Research Institute*, 42(3),137-151. https://doi.org/10.25020/je.2022.42.3.137.
[33]Shim, Do-Hyun,. (2022) The Main and Interaction Effect of Deep Emotional Labour and Supervisor's Emotional Support on Teacher-Child Emotional Interaction in Childcare Center Teachers. *The Korea Association of Child Care and Education*, 132, 55-76.https://doi.org/10.37918/kce.2022.1.132.55.
[34]Wang, H., Hall, N. C., & Taxer, J. L. (2019). Antecedents and consequences of teachers' emotional labor: A systematic review and meta-analytic investigation. *Educational Psychology Review,* 1e36. https://doi.org/10.1007/s10648-019-09475-3.
[35]Wei, S.D., Guan, J.F., Wang, S.M.. (2021). The Relationship between Preschool Teacher's Professional Identity, Emotional Labor and Professional Well-being. *China Journal of Health Psychology*, *29*(09):1367-1371. https://doi.org/10.13342/j.cnki.cjhp.2021.09.019.
[36]Wainwright, E., Marandet, E., Smith, F., & Rizvi, S.. (2010). The microgeographies of learning bodies and emotions in the 'classroom-salon'. *Emotion Space & Society, 3*(2), 80-89. https://doi.org/10.1016/j.emospa.2010.01.001.
[37]Xie, S., Wu, D., & Li, H.. (2022). The relationship between Chinese teachers' emotional labor, teaching efficacy, and young children's social-emotional development and learning. *Sustainability*, 14, 2205. https://doi.org/10.3390/su14042205.
[38]Yin, H., Huang, S., & Wang, W. (2016). Work environment characteristics and teacher well-being: The mediation of emotion regulation strategies. *International Journal of E*

nvironmental Research and Public Health, *13*(9), 907. https://doi.org/10.3390/ijerph13090907.

[39]Zhang, KS., Cui, XX., Wang, RD., Mu, CC., Wang, F.. (2022). Emotions, Illness Symptoms, and Job Satisfaction among Kindergarten Teachers: The Mediating Role of Emotional Exhaustion. *SUSTAINABILITY*, *14*(6), 3261. https://doi.org/10.3390/su14063261.

Click here to access/download
Supporting Information
Original data.sav

Click here to access/download
Supporting Information
Scale of Emotional Labor Ability for Kindergarten Teachers.doc
