# Supplementary material for: Development of emotional labor ability scale for kindergarten teachers
Source: PLoS One. 2025 Jun 23;20(6):e0325891. doi: 10.1371/journal.pone.0325891 (PMC12184924; doi:10.1371/journal.pone.0325891)
Supplement: Attachment — Submitted filename: Response to the Reviewers.docx [file pone.0325891.s014.docx]

**Report**

**Reviewer1** 09.03.2025

1.Several references (both in-text and in the list) are done not quite adequately: It should be Surname, initials and not full names [1,4,8,10,16, 17, 18,25, 26, 31, 32, 33]. Writing Grandey, & A. or Grandey, & Alice, as well as Beauchamp, & Catherine is incorrect. It should be Grandley, A.; Beauchamp, C.

**Thanks for the comment. Based on the comment, the results were included in the references**

[1][Byeon, H](https://www.webofscience.com/wos/alldb/general-summary?queryJson=%5b%7b)., [Yim, M](https://www.webofscience.com/wos/alldb/general-summary?queryJson=%5b%7b) S.. (2021). Mediating Effect of Resilience on the Relationship between Emotional Intelligence and Emotional Labor. Logos Management Review, 19(2), 97-114. https://doi.org/10.22724/LMR.2021.19.2.97.

[4]Cho,E., SUKYUNG, S.. (2022). Mediating Effect of Autonomy on the Relationship Between Self-leadership and Emotional Labor of Early Childhood Teachers. The Journal of Humanities and Social science, 13(6), 827-842. https://doi.org/10.22143/hss21.13.6.56.

[8]EUN,Y J. (2022). Analyze Factors to Influence the Turnover Intention of Childcare Teachers. The Journal of the Korea Contents Association, 22(11), 660-672. https://doi.org/10.5392/JKCA.2022.22.11.660.

[10]Grandey,A.. (2000). Emotion regulation in the workplace: a new way to conceptualize emotional labor. J Occup Health Psychol, 5(1), 95-110. https://doi.org/10.1037/1076-8998.5.1.95.

[16]Kyung,M Y.,Jang, H J.. (2021). Childcare Teacher’s Emotional Labor Clusters: The Differences of Inter-Personal Stress and Self-Esteem by Clusters. The Society of Convergence Knowledge Transactions, 9(1), 75-83. https://doi.org/10.22716/sckt.2021.9.1.008.

[17]Kwak,E., So-Hyun,J.. (2022). The Effect of Early Childhood Teacher’s Emotional Labor on Child-teacher Interaction: Serial Multiple Mediation of Teaching Efficacy and Burnout. Journal of Children’s Literature and Education, 23(3), 297-312. https://doi.org/10.22154/jcle.23.3.13.

[18][Kyung-chul,K](https://www.webofscience.com/wos/alldb/general-summary?queryJson=[{"rowBoolean":null,"rowField":"AU","rowText":"Kyung-chul, Kim"}]&eventMode=oneClickSearch).,Zhang[,X Y](https://www.webofscience.com/wos/alldb/general-summary?queryJson=[{"rowBoolean":null,"rowField":"AU","rowText":"Xinyu, Zhang"}]&eventMode=oneClickSearch) .., [Chunjing, Cai](https://www.webofscience.com/wos/alldb/general-summary?queryJson=[{"rowBoolean":null,"rowField":"AU","rowText":"Chunjing, Cai"}]&eventMode=oneClickSearch).. (2021). The Effect of Interpersonal Relationships in the Workplace of Kindergarten Teachers in China on Job Burnout: Focusing on the Emotional Intelligence Modulating Effect. International Journal of Early Childhood Education, 27(2), 89-115.

[25]No, S., Yeong-hee, K.. (2021) Effects of Preschool Teacher’s Emotional Labor on Burnout, Job Satisfaction, and Turnover Intention. Life Science Research Forum, 25(1), 69-81. https://doi.org/10.36357/johe.2021.25.1.69.

[26]Nan-Sil, K., Suhyun, K.. (2020). The Effects of Childcare Teachers’ Emotional Labor on Psychological Well-being. The Journal of Humanities and Social science, 11(5), 1659-1674. https://doi.org/10.22143/hss21.11.5.118.

[31][Shin, D](https://www.webofscience.com/wos/alldb/general-summary?queryJson=[{"rowBoolean":null,"rowField":"AU","rowText":"Shin, Dongju"}]&eventMode=oneClickSearch)., [Myoung, U S](https://www.webofscience.com/wos/alldb/general-summary?queryJson=[{"rowBoolean":null,"rowField":"AU","rowText":"Myoung, Um So"}]&eventMode=oneClickSearch).. (2020). A Study on the Relations among Emotional Labor Types, Perception of Emotional Display Rules and Emotion Regulation Strategies of Childcare Center Teachers. Korean Journal of Child Education and Care, 20(3),25-40. https://doi.org/10.21213/kjcec.2020.20.3.25.

[32][Seom, H S](https://www.webofscience.com/wos/alldb/general-summary?queryJson=[{"rowBoolean":null,"rowField":"AU","rowText":"Seom, Han San"}]&eventMode=oneClickSearch).. (2022). An Analysis on Kindergarten Teachers’ Emotional Labor. The Journal of EducationKyungin University of Education Educational Research Institute,42(3),137-151. https://doi.org/10.25020/je.2022.42.3.137.

[33]Shim, D H,. (2022) The Main and Interaction Effect of Deep Emotional Labour and Supervisor’s Emotional Support on Teacher-Child Emotional Interaction in Childcare Center Teachers. The Korea Association of Child Care and Education, 132, 55-76.https://doi.org/10.37918/kce.2022.1.132.55.

2.There are some minor spelling mistakes, e.g.: p. 29 the extend to which (should be the extent to which)

**Thanks for the comment. Based on the comment, Modified in the paper.**

“The extend to which” has been modified to“the extent to which”

3.There are some capitalization issues, e.g.: in the abstract "Methods" should be capitalized, in 2.2.4 questionnaire should be capitalized. Also in chapter 4 subtitles should be fully capitalized, not only the first words.

**Thanks for the comment. Thanks for the comment.**

I have made adjustments to the expression of this sentence in the text.

4. In section 3.1 the sentence "Conduct independent t-tests, ..." is in imperative mood, but it should be in Past Simple test (Independent t-tests were conducted, ..."

**Thanks for the comment.**

I have made adjustments to the expression of this sentence in the text.

Independent t-tests were conducted on the scores of the high and low groups for the 37 items, and subsequently, the Pearson correlation between each item and the total score was calculated.

**Report**

**Reviewer 2** 09.03.2025

1.The manuscript reports a study aiming to develop a scale titled Emotional Labor Ability Scale for Kindergarten Teachers.

Although the study certainly has its merits, such as adhering to protocols for scale development, it unfortunately contains a fundamental flaw: the lack of a conceptual or theoretical underpinning for the term “emotional labor ability.” The term “emotional labor ability” does not make sense. We can use terms like “emotional ability,” “emotional competence,” “emotional intelligence,” or “emotional skills,” but “emotional labor ability” is not appropriate. This is because emotional labor comprises several distinct constructs (i.e., surface acting, deep acting, and the expression of naturally felt emotions). The performance of these constructs cannot be aggregated into what might be termed an “ability.” For instance, a teacher who performs surface acting more does not necessarily have higher “emotional labor ability,” and the same applies to deep acting and the expression of naturally felt emotions. Throughout the literature on emotional labor, no researcher attempts to measure an “ability” related to emotional labor, despite the existence of numerous scales measuring emotional labor constructs. The absence of a scale measuring “emotional labor ability” is not a research gap but a logical reality; such an “ability” does not exist.

The authors claim that Grandey (2000) serves as the theoretical framework for the study. However, Grandey’s framework does not support the concept of “emotional labor ability.” Grandey’s framework is based on situational cues, emotional labor (deep acting, surface acting), and long-term consequences. In contrast, the “emotional labor ability” scale in this study includes emotional intelligence, the ability to internalize emotional labor rules, coordination in emotional labor, reflective ability after emotional labor, and the application of emotional labor strategies. Clearly, the five constructs of “emotional labor ability” in this study are not supported by existing literature.

I believe that this fundamental flaw alone renders the study illegitimate, regardless of how the manuscript is revised.

**Thanks for the comment. Based on the comment, The author makes the following explanation and makes some modifications in the article.**

**1 The Concept of "Emotional Labor Ability" Exists**

Firstly, it must be emphasized that "emotional labor ability" is not a non-existent concept. Rather, the term "emotional labor ability," as an academic concept, has been widely used by both domestic and international academic communities.

For instance, in the medical field, studies have indicated that possessing emotional labor ability is of great significance for establishing harmonious doctor-patient and nurse-patient relationships, as well as for improving patient satisfaction. **Having emotional labor ability is of great significance to build a harmonious doctor-patient relationship, nurse-patient relationship, and to improve patients' satisfaction**.^[^^[1]]^Moreover, research has also investigated the impact of narrative dialogue on the emotional labor capacity of male psychiatric nurses with lower levels of experience.**Previous studies have explored the impact of narrative dialogue on the emotional labor ability of low qualified male psychiatric nurses.**^[^^[2]]^

For the group of kindergarten teachers, scholars have emphasized the importance of conducting training programs aimed at enhancing their emotional labor ability^[^^[3]]^. Furthermore,A study in South Korea, based on positive psychology, **developed an emotional labor coping ability enhancement plan for preschool teachers and applied it to preschool teachers, achieving relatively positive experimental results.**^[^^[4]]^**Of particular importance is that enhancing the emotional labor ability of kindergarten teachers has become a fundamental consensus within the academic community both domestically and internationally**^[[5]][[6]][[7]][[8]]^**.**

**2 Review of Existing Research on the Structure of "Emotional Labor Ability"**

The concepts of surface acting, deep acting, and genuine emotion expression, as mentioned by experts, belong to "emotional labor strategies," which refer to the strategies or means employed by individuals in the process of emotional labor. These are not the structure of emotional labor itself.

Existing research has conducted preliminary explorations into the structural components of "emotional labor ability." In the early stages of research on emotional labor ability, some studies equated "emotional intelligence" with "emotional labor ability" based on the interpersonal interaction characteristics of emotional labor^[[9]]^, proposing that "emotional labor ability primarily encompasses aspects such as emotional perception ability, emotional understanding ability, emotional management ability, and emotional utilization ability."^[[10]][[11]][[12]]^

Specifically, focusing on the teacher population, researchers have indicated that teachers' emotional labor ability consists of three components: emotional perception ability, emotional regulation ability, and emotional expression ability^[[13]]^. Jennings et al. (2013)^[[14]]^ proposed that the emotional skills teachers need to acquire include affective skills, mindfulness awareness, and caring and compassion. Based on the aforementioned research findings, it can be inferred that emotional intelligence is a crucial psychological resource for individuals to navigate emotional labor ^[[15]]^. However, high emotional intelligence does not necessarily imply efficient emotional labor, as possessing a skill does not equate to successfully applying it.

As research on emotional labor ability progresses, researchers have begun to focus on exploring individuals' emotional labor ability within specific work contexts. It is believed that emotional labor ability should manifest as an individual's capacity to employ various emotional labor strategies, such as deep acting and genuine emotion expression, based on specific educational situations^[[16]][[17]][[18]]^. In the context of kindergarten teachers, existing research has summarized experiences and presented viewpoints on the conceptual connotation^[[19]]^, importance^[[20]]^, and improvement strategies of kindergarten teachers' emotional labor ability^[[21]]^. This provides valuable insights for further exploring the structural dimensions of kindergarten teachers' emotional labor ability.

**3 Theoretical Foundation and the Five Dimensions of "Emotional Labor Ability" Proposed in This Study**

The model of emotional labor mechanisms proposed by Grandey is closely linked to the five dimensions of "emotional labor ability" put forth in this study and provides a solid theoretical foundation for the development of the "Emotional Labor Ability Scale for Kindergarten Teachers."

Grandey's model divides the working mechanism of emotional labor into three stages: situational cues, emotional regulation processes, and long-term outcomes^[[22]]^. Specifically, Grandey emphasizes that during the stage of situational cues, individuals must first use emotional intelligence to perceive and understand the interaction expectations of work objects and the attributes of emotional events, and have a correct understanding and deep identification with the rules of emotional labor, to ensure that subsequent emotional expression behaviors conform to organizational requirements. This stage requires individuals to possess a certain level of emotional intelligence and the ability to internalize emotional labor rules.In the emotional regulation stage, guided by the rules of emotional labor, individuals use emotional labor strategies to regulate their external emotional expression behaviors and internal emotional experiences, transforming their subjective emotional experiences into social action forces that embody educational responsibilities. This stage requires individuals to have the ability to apply emotional labor strategies.After the completion of emotional labor, individuals need to reflect on the individual and organizational outcomes of emotional labor, including whether their emotional expression behaviors conform to professional behavioral norms and whether they contribute to achieving work objectives, in order to promote the improvement of their emotional labor abilities. This stage requires individuals to have the ability to reflect on emotional labor.Based on this, during the process of emotional labor, individuals need to possess emotional intelligence, the ability to internalize emotional labor rules, the ability to apply emotional labor strategies, and the ability to reflect on emotional labor.

Building upon this foundation, to explore the characteristics of emotional labor ability among kindergarten teachers, this study conducted behavioral event interviews with in-service kindergarten teachers to further supplement and refine the four dimensions of emotional labor ability derived from theoretical analysis. Through the analysis and synthesis of all interview content, it is preliminarily proposed that the emotional labor ability of kindergarten teachers comprises five dimensional elements: emotional intelligence, ability to internalize emotional labor rules, ability to apply emotional labor strategies, ability to collaborate in emotional labor, and ability to reflect on emotional labor.

Subsequently, this study conducted two rounds of expert consultations on the five dimensional elements and key competencies of kindergarten teachers' emotional labor ability. Based on the results of the expert consultations, this study initially constructed a preliminary model of kindergarten teachers' emotional labor ability, which includes five basic elements and 16 key competencies. Based on this model, the "*Initial Questionnaire on Kindergarten Teachers' Emotional Labor Ability*" was developed.

Lastly, a questionnaire survey was conducted among in-service kindergarten teachers, and the collected valid data were subjected to item analysis, exploratory factor analysis, confirmatory factor analysis, and reliability analysis. The results confirmed that the "*Emotional Labor Ability Scale for Kindergarten Teachers*" developed in this study exhibits good reliability and validity. The scale consists of 30 items, divided into five dimensions: emotional intelligence, ability to internalize emotional labor rules, ability to collaborate in emotional labor, ability to apply emotional labor strategies, and ability to reflect on emotional labor.

Existing research has not yet proposed the aforementioned five dimensions of emotional labor ability for kindergarten teachers, but this does not imply that the present study lacks literature support. From the perspective of the entire research process, the proposition of the five dimensions of emotional labor ability and the development of the scale in this study are grounded in Grandey's model of emotional labor process mechanisms as the theoretical foundation, and are constructed based on factual evidence from behavioral event interviews with kindergarten teachers, expert consultations, and questionnaire surveys. These constitute the significant innovations and unique value of this study.

**Report**

**Reviewer 3** 09.03.2025

1.Abstract:

Objective:

Ensure the research objectives are focusing on the scale development process to be aligned with the title.

Method:

Capital M

**Thanks for the comment. Based on the comment, Modified in the paper**

I have made structural adjustments to the abstract, and the revised abstract is as follows:

Kindergarten teachers' lack of emotional labor ability can lead to emotional exhaustion, burnout, identity alienation, and low job satisfaction. Systematic training is now an academic consensus. However, a suitable assessment instrument is lacking. This study aims to develop an emotional labor ability scale for kindergarten teachers and test its reliability and validity in China. First, based on the emotional labor working mechanism model, the dimensions of teachers' emotional labor ability were analyzed. Second, BEIs were conducted with 21 teachers to construct primary and secondary competency elements. Two rounds of expert consultation involving 5 experts revised these elements, resulting in an initial questionnaire. Finally, based on survey data from 818 teachers, various analyses (item, exploratory factor, confirmatory factor, reliability, convergence validity, and discriminant validity) were conducted. The scale has 30 items across 5 dimensions: emotional intelligence, internalizing emotional labor rules, coordination in emotional labor, reflection after emotional labor, and applying emotional labor strategies. These dimensions explain 71.757% of the variance. Confirmatory factor analysis shows good model fit (χ2/df=2.774, RMR=0.016, GFI=0.918, IFI=0.969, TLI=0.965, CFI=0.969, RMSEA=0.047). Internal consistency reliability coefficients range from 0.902 to 0.953. The scale is psychometrically sound and can effectively evaluate kindergarten teachers' emotional labor ability.

2.A vague problem statement to just mention 'room for in-depth exploration'.

The author(s) must ensure the problem statement highlights the lack of appropriate tools to measure emotional labor. What's the limitations of existing scales?

Provide citations and references. Page4

**Thanks for the comment. Based on the comment, , Modified in the paper.**

**Currently, there is no scale for** "*Emotional Labor Ability of Kindergarten Teachers*". At the same time, I have clearly marked the following paragraph in the text: For example, the research results on the structural dimensions of the emotional labor ability of kindergarten teachers based on actual teaching scenes have been found rare in number, and no existing research has been found on the tools for the evaluation of the emotional labor ability of kindergarten teachers.

3. 2.2.4 questionnaire survey	Page11

The author(s) may require to add details regarding how items for scale were generated.

Do also elaborate on the sampling process.

Before going into the Results, kindly provide a clear elaboration on what type of statistical methods used (eg Reliability Testing/Factor Analysis)

**Thanks for the comment. Based on the comment, Modified in the following.**

The development of the scale includes the following four steps:

2.2.1 Theoretical Framework; 2.2.2 Source of Initial Items; 2.2.3 Delphi Method; 2.2.4 Questionnaire Survey

In section 2.2.4 of the Questionnaire Survey, I added the following paragraph to introduce the analysis methods used: Overall, this study employed several analytical methods including item analysis, exploratory factor analysis, confirmatory factor analysis, reliability and Validity analysis.

4. 2.2.4 questionnaire survey	Page12

Provide enough details on participant recruitment details like sampling size and sampling criteria.

**Thanks for the comment. Based on the comment, Modified in the Following.**

The survey tool utilized in this study is the Initial Questionnaire of the Emotional Labor Ability of Kindergarten Teachers. This questionnaire was developed based on the results of previous literature research, interviews with kindergarten teachers, and expert consultations to ensure its content validity and relevance. The questionnaire comprises five dimensional elements and 37 items, with each question item adopting the Likert five-point scoring method to facilitate quantitative analysis.For participant recruitment, a convenience sampling method was employed to distribute the questionnaires. Specifically, the study targeted in-service kindergarten teachers in Hunan Province, China. The questionnaires were made available on the WJX.CN questionnaire network platform, which is a widely used and accessible online survey tool in the region.In terms of sampling size, a total of 818 pieces of valid data were collected from the in-service kindergarten teachers who participated in the survey. This sample size was deemed sufficient for conducting both exploratory and confirmatory factor analyses, which are essential steps in validating the questionnaire's structure and dimensionality.Regarding the sampling criteria, participants were selected based on their current employment status as kindergarten teachers in Hunan Province. No additional selection criteria were applied, such as age, gender, or years of teaching experience, to ensure a diverse and representative sample. The only requirement for participation was that the teachers were currently working in kindergartens and willing to complete the online questionnaire.After collecting the valid data, it was randomly split into two equal parts. The first 409 pieces of data were used for exploratory factor analysis (EFA), which aimed to identify the underlying factors or dimensions of the questionnaire. The other 409 pieces of data were reserved for confirmatory factor analysis (CFA), which was conducted to verify the factor structure identified in the EFA and assess the questionnaire's construct validity.

5. 3.Results	Page 12

The findings should be presented in detail. Any significant patterns or differences in the emotional labor abilities should be clearly explained.

**Thanks for the comment. Based on the comment, Modified in the Following.**

This study analyzed the meaning of each dimension of the scale and its position in the scale in the discussion section.

6.4. Discussion	Page 19

Could discuss on how this scale is practically applicable in real educational settings.

Could also make comparison with existing scales available by linking to the introduction (problem statement).

Thank you very much for your suggestion. This study focuses on analyzing the development and validation of the Emotional Labor Ability Scale for kindergarten teachers in the Chinese context, so the application effect of the scale in actual educational contexts has not been experimentally tested. Your suggestions provide the author with ideas for future research.

In addition, due to the lack of an emotional labor capacity scale for kindergarten teachers, it is not possible to compare it with existing scales.

7. 4. Discussion	Page 19

The discussion should be organised around the research objectives.

**Thanks for the comment. Based on the comment,, Modified in the paper.**

The aim of this study is to develop a scale for measuring the emotional labor capacity of kindergarten teachers and verify its reliability and validity in the context of Chinese education. Therefore, in the discussion section, we focused on the structure of the scale, namely analyzing the five dimensions of the scale, the important position of each dimension in the overall scale, and the items under each dimension.

8. 5. Limitations and Future Research	Page 26

Lacked of practical implications and recommendations. There are no references here where the write-up looks much of opinion-based.

**Thanks for the comment. Based on the comment,, Modified in the paper.**

The theoretical and practical significance of the research were added after the discussion:

This study has certain theoretical significance. First, the five-factor structure of the emotional labor ability of kindergarten teachers constructed in this study is basically consistent with the working mechanism model of emotional labor proposed by Grundi, which helps to demonstrate the explanatory power of the working mechanism model of emotional labor in the field of education. Secondly, previous studies mainly focused on one aspect of emotional labor ability, such as emotional intelligence, application ability to emotional labor strategy, etc. Yet this study conducted a systematic and comprehensive analysis on the constituent elements of the emotional labor ability of kindergarten teachers, and proposed a five-factor model of the emotional labor ability of kindergarten teachers, which can enrich the connotation of the emotional labor ability of kindergarten teachers. Finally, most of the existing studies use a single speculative research method to describe the components of the emotional labor ability of kindergarten teachers. Thus it remains unknown whether the deductive reasoning by personal perceptual experience can withstand data verification, which leads to a relative lack of feasibility and applicability of research conclusions. This study used mixed research methods to verify and analyze the internal structure of the emotional labor ability of kindergarten teachers, and developed the Emotional Labor Ability Scale for Kindergarten Teachers that meets the requirements of psychological measurement, which has certain practicality and can be provided as an effective tool for the development of quantitative research in this field in the future.

This study has certain practical significance, too. The Emotional Labor Ability Scale for Kindergarten Teachers developed in this study can be used by kindergarten managers for the evaluation of the emotional labor ability of applicants, so as to change the situation where kindergarten teacher recruitment only focuses on the teaching ability of kindergarten teachers and ignores their emotional emotional ability. In doing so, job seekers who do not match the kindergarten-teacher career can be eliminated from the start to maximize the personnel and job matching of kindergarten teachers so that the stability of the kindergarten teacher team can be effectively promoted. Additionally, the scale is also an evaluation tool for the effectiveness of training on the emotional labor ability of kindergarten teachers. The measurement can be conducted before and after the training to judge whether it is effective and to ensure the most scientific and effective training methods are adopted so that the post-service training resources can be efficiently coordinated.

9. 6. Conclusion	Page 27

The conclusion seems to be a little weak and incomplete.

**Thanks for the comment. Based on the comment, , Modified in the following**

The Emotional Labor Ability Scale for Kindergarten Teachers, developed through rigorous research in this study, comprises five comprehensive dimensional elements. These dimensions include emotional intelligence, the proficiency in internalizing emotional labor rules, the coordination ability essential for managing emotional labor effectively, the practical application ability to implement emotional labor strategies, and the reflective ability that follows emotional labor experiences. These dimensions are further broken down into 15 distinct competency elements, which are assessed through a total of 30 meticulously crafted items.The scale has undergone a thorough validation process, encompassing item analysis, exploratory factor analysis, confirmatory factor analysis, reliability analysis, convergence validity analysis, and discriminant validity analysis. The results from these analyses consistently demonstrate that the scale possesses robust psychometric properties. It exhibits high reliability, indicating consistent and stable measurements over time. Furthermore, the scale demonstrates strong convergence validity, confirming that it accurately measures the intended constructs related to emotional labor ability in kindergarten teachers. Additionally, the discriminant validity analysis confirms that the scale distinguishes between different constructs, ensuring its specificity and precision.

1. ^[]^Juan Z,et,al.. (2020). Application and prospect of emotional labor in nursing research. *Chinese Nursing Management.* 20(10):1528-1533.
2. ^[]^Lingfang, Z. , Jianying, W. , & Donghong, Z. . (2019). Effect of narrative dialogue on emotional labor ability in male psychiatric nurses with low seniority. *Journal of Psychiatry*.
3. ^[]^[Cho, E](https://www.webofscience.com/wos/alldb/general-summary?queryJson=[{"rowBoolean":null,"rowField":"AU","rowText":"Cho,, Eunjung"}]&eventMode=oneClickSearch)., [SUKYUNG, S](https://www.webofscience.com/wos/alldb/general-summary?queryJson=[{"rowBoolean":null,"rowField":"AU","rowText":"SUKYUNG, SHIN"}]&eventMode=oneClickSearch).. (2022). Mediating Effect of Autonomy on the Relationship Between Self-leadership and Emotional Labor of Early Childhood Teachers.The Journal of Humanities and Social science, 13(6):827-842.
4. ^[]^On, E.. (2022). The Development and Effect of Positive Psychology-based Managing Skill Improvement Program for Emotional Labors of Kindergarten Teachers. *The Journal of Learner-Centered Curriculum and Instruction*. 22(19): 217-231.
5. ^[]^[No, S k](https://www.webofscience.com/wos/alldb/general-summary?queryJson=%5b%7b)., [Yeong-hee, K.](https://www.webofscience.com/wos/alldb/general-summary?queryJson=%5b%7b). (2021) Effects of Preschool Teacher’s Emotional Labor on Burnout, Job Satisfaction, and Turnover Intention. *Life Science Research Forum, 25*(1), 69-81. DOI:10.36357/johe.2021.25.1.69.
6. ^[]^Peng, J., He, Y., Deng, J., Zheng, L., Chang, Y., & Liu, X.. (2019). Emotional labor strategies and job burnout in preschool teachers: psychological capital as a mediator and moderator. *Journal of neurosurgical sciences*(3), 63. DOI:10.3233/WOR-192939.
7. ^[]^[EUN, Y J](https://www.webofscience.com/wos/alldb/general-summary?queryJson=%5b%7b). (2022). Analyze Factors to Influence the Turnover Intention of Childcare Teachers. *The Journal of the Korea Contents Association, 22*(11), 660-672. DOI:10.5392/JKCA.2022.22.11.660.
8. ^[]^Wei S D., Guan J F., Wang S M., Liang J Q..(2021) The relationship between professional identity, emotional labor, and professional well-being among kindergarten teachers [J]. *China Journal of Health Psychology*,, 29(09): 1367-1371.
9. ^[]^Mayer, J. D., & Salovey, P.. (1993). The intelligence of emotional intelligence. Imagination Cognition and Personality, 17(3), 433-442. DOI:10.1016/0160-2896(93)90010-3.
10. ^[]^Mayer, J. D., Salovey, P., Caruso, D. R., & Sitarenios, G.. (2003). Measuring emotional intelligence with the msceit v2.0. Emotion, 3(1), 97-105. DOI:10.1037/1528-3542.3.1.97.
11. ^[]^Mayer, J. D., Salovey, P., & Caruso, D. R. (2004). Emotional intelligence: Theory, findings, and implications. Psychological Inquiry, 15(3), 197e215. https://doi.org/10.1207/s15327965pli1503_02.
12. ^[]^Jennings, P. A., & Greenberg, M. T. (2009). The prosocial classroom: Teacher social and emotional competence in relation to student and classroom outcomes. Review of Educational Research, 79(1), 491e525. https://doi.org/10.3102/0034654308325693.
13. ^[]^Rindermann, H. (2009). Ekf: Emotionale-Kompetenz-Fragebogen. Hogrefe.
14. ^[]^Jennings, P. A., Frank, J. L., Snowberg, K. E., Coccia, M. A., & Greenberg, M. T. (2013). Improving classroom learning environments by Cultivating Awareness and Resilience in Education (CARE): Results of a randomized controlled trial. School Psychology Quarterly, 28(4), 374e390. https://doi.org/10.1037/spq0000035.
15. ^[]^Wong, C. S., & Law, K. S.. (2002). The effects of leader and follower emotional intelligence on performance and attitude: an exploratory study. Leadership Quarterly, 13(3), 243-274.DOI:10.1016/S1048-9843(02)00099-1.
16. ^[]^Wang, H., Hall, N. C., & Taxer, J. L. (2019). Antecedents and consequences of teachers' emotional labor: A systematic review and meta-analytic investigation. Educational Psychology Review, 1e36. https://doi.org/10.1007/s10648-019-09475-3.
17. ^[]^Chang, M.-L., & Taxer, J. (2020). Teacher emotion regulation strategies in response to classroom misbehavior. Teachers and Teaching, 1e17. https://doi.org/10.1080/13540602.2020.1740198.
18. ^[]^Yin, H., Huang, S., & Wang, W. (2016). Work environment characteristics and teacher well-being: The mediation of emotion regulation strategies. International Journal of Environmental Research and Public Health, 13(9), 907. https://doi.org/10.3390/ijerph13090907.
19. ^[]^[Hong](https://www.webofscience.com/wos/alldb/general-summary?queryJson=[{"rowBoolean":null,"rowField":"AU","rowText":"seonghee, Hong"}]&eventMode=oneClickSearch), S.. (2018). The Study on the Teacher's Emotional Labor in Early Childhood Education Institution. *DONGAINMUNHAK,* 45,473-494.DOI:10.52639/JEAH.2018.12.45.473.
20. ^[]^[Zhang, KS](https://www.webofscience.com/wos/author/record/32443055)., [Cui, XX](https://www.webofscience.com/wos/author/record/7010875)., [Wang, RD](https://www.webofscience.com/wos/author/record/37318373)., [Mu, CC](https://www.webofscience.com/wos/author/record/25645927)., [Wang, F](https://www.webofscience.com/wos/author/record/17501436).. (2022). Emotions, Illness Symptoms, and Job Satisfaction among Kindergarten Teachers: The Mediating Role of Emotional Exhaustion. *SUSTAINABILITY, 14*(6), 3261. DOI:10.3390/su14063261.
21. ^[]^[Seom, H S](https://www.webofscience.com/wos/alldb/general-summary?queryJson=[{"rowBoolean":null,"rowField":"AU","rowText":"Seom, Han San"}]&eventMode=oneClickSearch).. (2022). An Analysis on Kindergarten Teachers’ Emotional Labor. *The Journal of EducationKyungin University of Education Educational Research Institute,42*(3),137-151. DOI:10.25020/je.2022.42.3.137.
22. ^[]^Grandey, A. (2000). Emotional regulation in the workplace: a new way to conceptualize emotional labor. *Journal of Occupational Health Psychology, 5*(1), 95-110. DOI:10.1037/1076-8998.5.1.95.
