# Supplementary material for: Development of emotional labor ability scale for kindergarten teachers
Source: PLoS One. 2025 Jun 23;20(6):e0325891. doi: 10.1371/journal.pone.0325891 (PMC12184924; doi:10.1371/journal.pone.0325891)
Supplement: Attachment — Submitted filename: Response_to_the_Reviewers_auresp_2.docx [file pone.0325891.s015.docx]

**Report**

**Additional Editor Comments** 16.04.2025

Abstract

1.Please expand "BEIs" to its full form in the abstract for clarity, especially for readers unfamiliar with the acronym.

**Thanks for the comment. Based on the comment, I have made the following modifications to the abstract:**

Second, Behavioral Event Interview were conducted with 21 teachers to construct primary and secondary competency elements.

Introduction

2.Revise lines 7-10 in the second paragraph for clarity and conciseness.

**Thanks for the comment. Based on the comment, I have condensed the content of the paper:**

In terms of the teacher group, some researchers have pointed out that teachers' emotional labor ability includes three components: emotional perception ability, emotional regulation ability, and emotional expression ability.

3.The introduction discusses multiple related constructs (emotional labor, emotional intelligence, emotional labor ability) but does not clearly differentiate them. Please provide a more precise definition of "emotional labor ability" early on to strengthen conceptual clarity.

**Thanks for the comment. Based on the comment, I have added the definition of "emotional labor ability of kindergarten teachers" in the corresponding position of the introduction:**

Based on existing research results, this study believes that the emotional labor ability of kindergarten teachers refers to the ability of kindergarten teachers to actively regulate their internal emotional experiences and external emotional expressions according to the professional requirements of kindergarten teachers in the process of education and teaching, in order to complete educational and teaching tasks.

4.Clarify why existing tools are inadequate or unsuitable for kindergarten teachers specifically in the introduction.

**Thanks for the comment. Based on the comment, I have provided explanations in the corresponding position of the introduction:**

Based on the suggestion, I have added the following content to the introduction section of the article:

The existing research results have provided useful references for us to understand the concept of emotional labor ability of kindergarten teachers. However, we need to recognize that the existing research mainly adopts speculative research methods to empirically explore the constituent elements of emotional labor ability of kindergarten teachers, and has not yet adopted empirical research methods to systematically verify and demonstrate the structural dimensions of emotional labor ability of kindergarten teachers. Based on this, there is still significant room for exploration in terms of theoretical appropriateness and practical explanatory power of existing research conclusions on the emotional labor ability of kindergarten teachers. This research gap provides an important opportunity for theoretical innovation and methodological breakthroughs in this study.

5.Some sections, particularly the discussion of emotional intelligence, could be more concise. Maintain focus on emotional labor ability as a distinct construct.

**Thanks for the comment. Based on the comment, I have removed this paragraph from the introduction section of the article:**

Based on the above research results, it is speculated that emotional intelligence is an important psychological resource for individuals to cope with emotional labor, but high emotional intelligence does not necessarily mean efficient emotional labor, because having ability is not equivalent to being able to successfully apply it.

Methods

6.Statistical Analysis Details: Provide a detailed description of the statistical methods used, including the software, tests, assumptions, treatment of outliers, missing data handling, and sample size calculations. This will improve transparency and reproducibility.

**Thanks for the comment. Based on the comment, I have added an explanation of the analysis software in the corresponding section of the methods：**

After collecting valid data, use SPSS software to randomly split the data into two parts.

7.Participant Demographics: Include detailed demographic information about the participants (age, gender distribution, years of experience, etc.). This will clarify the sample characteristics and support generalizability.

Thank you for your careful review and valuable suggestions on this article. We fully agree with the importance of your request to supplement demographic variable characteristics. Demographic information does help to more comprehensively evaluate the applicability of the scale among different teacher groups and provide more targeted references for subsequent research.

In this study, our primary goal was to develop and validate the psychometric characteristics of the scale (such as construct validity and reliability), therefore demographic data was not systematically collected. This design choice is mainly based on two considerations: (1) focusing on the validity testing of the core construct of the scale; (2) Control the length of the questionnaire to improve the completion rate of teachers' responses. We acknowledge that the lack of demographic variables does limit the analysis of group differences, but it should be noted that the current reliability and validity test results of the scale are based on representative overall data of the teacher population, and their scientific validity is not affected.

**To actively respond to your suggestions, we plan to take the following improvement measures in research:**

In the Limitations and Future Research section of this article, we provide an explanation of the current limitations and suggest that future research further explore the impact of demographic characteristics on emotional labor capacity.

Fourth, In this study, our primary goal was to develop and validate the psychometric characteristics of the scale (such as construct validity and reliability), therefore demographic data was not systematically collected. In the next stage of the large sample survey, we will include core demographic indicators (such as age, teaching experience, education, kindergarten type, etc.) and analyze the relationship between these factors and scale scores.

support generalizability

8.Data Availability Statement: Include a data availability statement specifying where the data will be made available, following PLOS ONE’s data sharing policies.

**Thanks for the comment. This study mainly used data obtained from a questionnaire survey, which has been included in the "Supporting Information" file in the form of an Excel spreadsheet. When submitting the revised paper, it was also submitted to the submission system for readers to use.**

**In addition, I provided the following explanation in my paper:**

**Data availability**

The data used in this study has been included in the 'Supporting Information' file and can be published together with the paper for readers to use.

9.Provide an ethics approval number or reference ID to comply with PLOS ONE’s ethical standards.

**Thanks for the comment. I have indicated the ethical approval number in the article and submitted the original ethical approval document as an attachment to the system for your reference.**

The ethical approval number is 2023255.

10.Ensure consistency in capitalization in Figure 1. Standardize terms and follow PLOS ONE’s formatting rules for figures.

**Thanks for the comment. Based on the comment, I have adjusted the capitalization of the text in the image based on the suggestions and uploaded the relevant images as attachments.**

11.The sample of 4 female teachers and 1 male teacher may not be sufficient to explore the unique qualities of emotional labor ability. Could you explain how this small sample was justified?

**Thanks for the comment.**

Based on the advice of the previous external audit expert, we have supplemented the interviews with 18 female teachers and 3 male teachers. We have updated the information in the abstract, but we forgot to update the interviewee's information here. Please forgive our negligence. I have now made a correction in the article:

Among them, there were 18 female teachers and 3 male teacher.

12.For the Delphi Method, please provide the characteristics (age, gender, specialization, experience, etc.) of the experts involved.

**Thanks for the comment. Based on the comment, I inserted a table in the corresponding position of the article listing all the information of the experts.**

Their demographic variable characteristics are shown in Table 1.

| Table 1 Demographic Characteristics of the Experts | | |
| --- | --- | --- |
| Characteristics | Frequency (N) | Proportion (%) |
| **Age (years)** |  |  |
| 30-40 | 1 | 20% |
| 40-50 | 3 | 60% |
| ≥50 | 1 | 20% |
| **Highest educational degree** |  |  |
| College | 0 | 0% |
| Undergraduate | 1 | 20% |
| Master’s and doctorate | 4 | 80% |
| **Title** |  |  |
| Junior professional title | 1 | 20% |
| Senior professional title | 2 | 40% |
| Associate senior professional title | 2 | 40% |
| **Work experience (years)** |  |  |
| 11–15 | 3 | 60% |
| 15–20 | 1 | 20% |
| ≥20 | 1 | 20% |
| **Research field** |  |  |
| Kindergarten teacher education | 2 | 40% |
| Kindergarten Teacher Management | 2 | 40% |
| Basic Theory of Preschool Education | 1 | 20% |

13.Figure 2: Use title case for all headings/subheadings (e.g., "Emotional Recognition" instead of "emotional recognition") to follow academic conventions.

**Thanks for the comment. Based on the comment, I have adjusted the letters in the image and uploaded it as an attachment.**

14.Questionnaire Survey: The first 4 lines of the survey are duplicated. Please remove the redundancy.

**Thanks for the comment. Based on the comment, I have already deleted the corresponding position in the text as requested.**

Sampling and Representativeness

15.Ensure the sample accurately reflects the broader population of kindergarten teachers. The convenience sampling method was used, please clarify how this may impact the generalizability of the results.

**Thanks for the comment. Based on the comment, I have provided the following explanation at the corresponding position in the text:**

Second, Due to my limited personal abilities, I used convenience sampling to conduct a questionnaire survey and reliability and validity testing only among kindergarten teachers in Hunan Province, China. The sample size may not represent the different urban and rural areas and regions of kindergarten teachers in China, and future research needs to further expand the survey population.

16.Clarify whether any additional criteria (e.g., age, gender, years of experience) were considered in participant selection. How did you ensure diversity and representativeness in the sample for scale development?

**Thanks for the comment.**

During the research process, I made efforts to collect more questionnaires, but did not screen demographic variables such as age, gender, and teaching experience of the participants. Instead, I conducted reliability and validity tests on the data after the survey to ensure that the scale had good psychological measurement characteristics.

17.Although 818 valid responses were collected, more information on how the sample size was determined and whether statistical power analysis was conducted for factor analyses would be helpful. Was the sample size adequate for these analyses, and how does it support the scale’s validity?

**Thanks for the comment. Based on the comment, I have provided the following explanation on the issue of sample size in the relevant section of the paper:**

Previous studies have suggested that the number of valid questionnaires should be at least 10 times the number of items on the scale to be more reliable (He, 2025). The number of valid questionnaires in this study is 22.1 times the total number of questions, which is much larger than the theoretical sample threshold (37 × 10). Therefore, it meets the sample conditions of the questionnaire survey and reflects the richness of the data volume.

Demographics and Scale Details

18. Provide more information on participants’ demographics (e.g., gender, age, years of experience, educational background). This will help assess whether the sample is diverse enough for developing a valid Emotional Labor Ability Scale.

Thank you for your careful review and valuable suggestions on this article. We fully agree with the importance of your request to supplement demographic variable characteristics. Demographic information does help to more comprehensively evaluate the applicability of the scale among different teacher groups and provide more targeted references for subsequent research.

In this study, our primary goal was to develop and validate the psychometric characteristics of the scale (such as construct validity and reliability), therefore demographic data was not systematically collected. This design choice is mainly based on two considerations: (1) focusing on the validity testing of the core construct of the scale; (2) Control the length of the questionnaire to improve the completion rate of teachers' responses. We acknowledge that the lack of demographic variables does limit the analysis of group differences, but it should be noted that the current reliability and validity test results of the scale are based on representative overall data of the teacher population, and their scientific validity is not affected.

To actively respond to your suggestions, we plan to take the following improvement measures in future research:

In the next stage of the large sample survey, we will include core demographic indicators (such as age, teaching experience, education, kindergarten type, etc.) and analyze the relationship between these factors and scale scores.

In the discussion section of this article, we will provide an explanation of the current limitations and suggest that future research further explore the impact of demographic characteristics on emotional labor capacity.

developing a valid Emotional Labor Ability Scale.

19.Please specify how many items each of the five dimensions in the questionnaire contains.

**Thanks for the comment. Based on the comment, I have provided the following explanations in the relevant sections of the article:**

Emotional intelligence refers to the ability of kindergarten teachers to recognize and understand the emotional state of others in interpersonal interaction, and to use this information to guide the whole process of emotional labor. It includes two sub-dimensions, namely the ability of emotional recognition and the ability of emotional understanding, and a total of 8 items.

The ability of internalizing emotional labor rules refers to the ability of kindergarten teachers to deeply identify with and consciously practice the code of conduct of emotional labor for the normal operation of emotional labor, including the subconsciousness of emotional labor rules and the automation of emotional labor rules, with a total of 5 items.

The coordination ability in emotional labor refers to the ability of kindergarten teachers to deal with emotional events in complex educational situations while ensure the smooth progress of all aspects of the kindergarten's daily activities. It mainly includes six sub-dimensions, namely the ability to coordinate collective teaching activities of the kindergarten, the ability to coordinate the morning activities of children, the ability to coordinate the life activities of children, the ability to coordinate the activities in corresponding activity area, and the ability to coordinate the outdoor or after-school activities of children, totaling 6 items.

The application ability to emotional labor strategies refers to the ability of kindergarten teachers to use certain emotional labor strategies according to the needs of educational situations in the process of emotional labor, including two sub-dimensions, namely the ability to use deep performance strategy and the ability to use natural performance strategy, with a total of 6 items.

The essence of reflective ability after emotional labor is that kindergarten teachers actively review their own emotional labor process to find their own problems in emotional labor and to find effective ways to solve problems, including three sub-dimensions, namely reflective perseverance, the ability to reflect on relevant theoretical knowledge and the ability to reflect on emotional labor experience, with a total of 8 items.

20.Clarify whether the questionnaire includes negatively worded items that require reverse scoring and explain how these items are handled in the final scoring process. This will ensure transparency in how emotional labor ability is quantified.

**Thanks for the comment.**

There was no reverse scoring question designed in this study. The reason is that there is still controversy over the impact of reverse questions on the reliability and validity of tests. For example, Weems et al. found that using a mix of positive and negative questions would lower the reliability of the test^[1]^. Sandoval and Lambert found that mixing forward questions with reverse questions in teacher assessment can improve reliability and validity^[2]^. Schriesheim and Hill found that using reverse questions can improve test validity but not reliability among college students^[3]^.

Results
21.The authors have not provided information regarding participant scores. This could be an oversight, as such details are crucial for understanding the results and ensuring transparency in the data analysis process. Please clarify how participant scores were handled and presented.

**Thanks for the comment. Based on the comment, I have provided the following explanations in the relevant sections of the article:**

The total score of each subject on the scale was calculated and then ranked in ascending order. The top 27% were the low-score groups while the bottom 27% were the high-score groups. Independent t-tests were conducted on the scores of the high and low groups for the 37 items, and subsequently, the Pearson correlation between each item and the total score was calculated.

22.In the Excel spreadsheet, it is unclear what "Q59" refers to, as the manuscript states that the questionnaire consists of 37 items across five dimensions. Could the authors clarify whether this is an error, an omitted item, or if additional questions were used but not reported in the manuscript?

I am very sorry that my carelessness has caused inconvenience to your reading. While designing and developing this scale, I also investigated research questions in other fields. However, when I submitted the raw data, I forgot to delete all other variable information and submitted it all. **I have updated the data this time and submitted it as' supporting information 'to the system.**

23.It would be beneficial to include a copy of the questionnaire with the manuscript, either as a supplementary file or by presenting all items in full within a table. This would enhance transparency and allow readers to assess the scale's content and structure.

**Thanks for the comment. Based on the comment, I will submit a copy of the questionnaire as a supplementary document.**

| Copy of the Emotional Labor Ability Scale for Kindergarten Teachers | |
| --- | --- |
|  | Question items |
| 1 | I can judge a child's emotional state through their facial expressions |
| 2 | I can judge a child's emotional state by their tone of speech |
| 3 | I can judge a child's emotional state through their body movements |
| 4 | I can judge a child's emotional state through their physiological arousal (shortness of breath, tremors, etc.) |
| 5 | I can judge the compound emotions of young children (such as anger, restlessness, surprise, etc.) by observing their words and expressions |
| 6 | I can understand why young children feel happy |
| 7 | I can understand why young children feel scared |
| 8 | I can understand why young children feel angry |
| 9 | For me, the normative requirements that kindergarten teachers need to follow when expressing their emotions are not unfamiliar |
| 10 | I strongly agree with the normative requirements that kindergarten teachers should follow when expressing emotions, such as gentleness, kindness, enthusiasm, etc |
| 11 | My attitude towards the normative requirements that kindergarten teachers should follow in expressing their emotions and behaviors is relatively stable |
| 12 | I firmly believe that the emotional expression behavior of kindergarten teachers must follow certain normative requirements |
| 13 | I strongly advocate that kindergarten teachers must follow certain normative requirements when expressing their emotions |
| 14 | When interacting with young children, if I need to show excitement, I can feel it from the bottom of my heart |
| 15 | When interacting with young children, if I need to show enthusiasm, I can feel it from the bottom of my heart |
| 16 | When interacting with young children, if I need to show a relaxed mood, I can feel it from the bottom of my heart |
| 17 | When interacting with young children, I can naturally show a sad emotional state to meet work needs (such as reaching empathy with young children) |
| 18 | When interacting with young children, I can naturally show an angry emotional state to meet work needs (such as expressing negation of problematic behavior in young children) |
| 19 | When interacting with young children, I can naturally show a tense emotional state to meet work needs (such as attracting children's attention, etc.) |
| 20 | When dealing with emotional events, I can still ensure the smooth progress of children's morning activities |
| 21 | When dealing with emotional events, I can still ensure the smooth progress of collective teaching activities for young children |
| 22 | When dealing with emotional events, I can still ensure the smooth progress of children's daily activities (such as eating, drinking, washing, toileting, sleeping, leaving the kindergarten, etc.) |
| 23 | When dealing with emotional events, I can still ensure the smooth progress of activities in the children's activity area |
| 24 | When dealing with emotional events, I can still ensure the smooth progress of outdoor activities for young children |
| 25 | When dealing with emotional events, I can still ensure the smooth progress of children's activities outside the kindergarten |
| 26 | I can reflect on the rationality of my emotional expression behavior based on my experience of emotional interaction with young children |
| 27 | I can reflect on the rationality of my emotional expression behavior based on my own emotional expression experience |
| 28 | I can reflect on whether my emotional expression behavior contributes to the development of children's emotional intelligence based on relevant theoretical knowledge |
| 29 | I can reflect on whether my emotional expression behavior helps meet the emotional needs of young children based on relevant theoretical knowledge |
| 30 | I can reflect on whether my emotional expression behavior meets the emotional requirements of kindergarten regulations based on relevant theoretical knowledge |
| 31 | I can persistently reflect on whether my emotional expression and behavior are reasonable |
| 32 | When my mood is relatively low, I can still persist in reflecting on whether my emotional expression behavior is reasonable |
| 33 | To make my emotional expression more in line with expectations, I am able to actively reflect on my emotional expression behavior on a continuous basis |

**Report**

**Reviewer 1** 16.04.2025

1.Beauchamp, & Catherine. (2015). Reflection in teacher education: issues emerging from a review o f current literature. Reflective Practice, 16(1), 123-141. [https://doi.org/10.1080/14623943.2014.98252](https://doi.org/10.1080/14623943.2014.98252)5.
should be written as
[2]Beauchamp, C. (2015). Reflection in teacher education: issues emerging from a review of current literature. Reflective Practice, 16(1), 123-141.[https://doi.org/10.1080/14623943.2014.98252](https://doi.org/10.1080/14623943.2014.98252)5.

**Thank you for your suggestion. I have updated it in the references section**.

**Report**

**Reviewer 2** 16.04.2025

1.Thank you for addressing all the required issues from the first review. In order to make the article clearer, the authors should provide a clearer comparison with existing emotional labour-related scales and their limitations to address the lack of appropriate tools for measuring EL in kindergarten teachers.

In the practical implication, a brief discussion on how the scale could be integrated into actual kindergarten teacher training programmes and how its effectiveness could be tested in future studies.

**Thanks for the comment. Based on the comment, I have made the following additions in the corresponding positions in the text:**

The existing research results have provided useful references for us to understand the concept of emotional labor ability of kindergarten teachers. However, we need to recognize that the existing research mainly adopts speculative research methods to empirically explore the constituent elements of emotional labor ability of kindergarten teachers, and has not yet adopted empirical research methods to systematically verify and demonstrate the structural dimensions of emotional labor ability of kindergarten teachers. Based on this, there is still significant room for exploration in terms of theoretical appropriateness and practical explanatory power of existing research conclusions on the emotional labor ability of kindergarten teachers. This research gap provides an important opportunity for theoretical innovation and methodological breakthroughs in this study.

In addition, this scale can be used as an evaluation tool for the effectiveness of emotional labor ability training for kindergarten teachers. The emotional labor ability of trainees can be measured before and after the training activities. By comparing the scores of kindergarten teachers at different stages of the training activities, the effectiveness of emotional labor ability training can be judged, and the most scientific and effective training methods can be selected to efficiently coordinate post service training resources for kindergarten teachers.

**Report**

**Reviewer 3** 16.04.2025

In general, the authors have coped with the task of developing a scale of Emotional Labor Ability of Kindergarten Teachers and proposed their own version of operationalisation for the Emotional Labor construct and developed their scale in accordance with it. Of course, it would be possible to discuss the operationalisation option. For example, why only such components of emotional intelligence as recognition and understanding are considered as individual factors, but such component as empathy is not included. However, in this case we can agree with the authors' understanding of this construct.
Psychometric verification of the developed scale was carried out quite correctly, but the results of confirmatory analysis, resulting in the proposed five-factor model, do not seem to be complete, as the authors present (Table 3) fit indices only for one- and five-factor models. T he question about the quality of models with other number of factors (2, 3 ,4?) remains unanswered. If the authors had presented data on the basis of which the readers would have been convinced of the advantage of the five-factor model, then the work of the researchers would have been completed and substantiated.
As for the correctness of the use of language, it is mostly correct, however, I am not sure about the capital W in the sentence ‘...that is, When there is a certain gap between the kindergarten teachers’ inner emotional feelings ....’ (p.18).

**Thanks for the comment. Based on the comment, I have made the following additions in the corresponding positions in the text:**

①Table 4(Fitting Indicator of the Model of the Emotional Labor Ability of Teachers) mainly aims to illustrate the following points:

To establish an optimal model, multiple validations were conducted on the model(see Table 4) and it is found that the χ2/df, GFI, IFI, TLI, CFI, and RMSEA in one factor model did not reach the recommended values, while all indicators of the five-factor model met the recommendation requirements. This indicated that the five-factor model fits well and is in line with the actual situation.

Meanwhile, I have provided the original data in the attachment as a reference.

②

that is, When there is a certain gap between the true emotional feelings of kindergarten teachers and the emotional labor rules stipulated by the organization, kindergarten teachers will use surface acting strategies to cope. This means that kindergarten teachers only change their external emotional expression to meet organizational requirements, but their inner selves do not fully agree with the laws of emotional labor, which can lead to emotional disorders, emotional exhaustion, professional burnout, and other situations among kindergarten teachers (Hülsheger et al., 2010).

1. Weems, G. H., & Onwuegbuzie, A. J. (2001). The impact of midpoint responses and reverse coding on survey data. Measurement & Evaluation in Counseling & Development, 34(3), 166-176.
2. Sandoval, Jonathan|Lambert, Nadine M. (1978). Reliability and validity of teacher rating procedures in the assessment of hyperactivity as a function of rating scale format. Behavior Rating Scales, 30.
3. Schriesheim, C. A., & Hill, K. D. (1981). Controlling acquiescence response bias by item reversals: the effect on questionnaire validity. Educational & Psychological Measurement, 41(4), 1101-1114.
