# Supplementary material for: Development of emotional labor ability scale for kindergarten teachers
Source: PLoS One. 2025 Jun 23;20(6):e0325891. doi: 10.1371/journal.pone.0325891 (PMC12184924; doi:10.1371/journal.pone.0325891)
Supplement: Attachment — Submitted filename: Response_to_the_Reviewers_auresp_3.docx [file pone.0325891.s016.docx]

**Report**

**Additional Editor Comments** 30.04.2025

Comment 16: Clarify whether any additional criteria (e.g., age, gender, years of experience) were considered in participant selection. How did you ensure diversity and representativeness in the sample for scale development?
While the authors' response is methodologically acceptable in focusing on psychometrics, the absence of demographic information limits the generalizability and interpretability of the scale. The explanation does not fully address the issue of sample representativeness. Since the construct relates to emotional labor in teachers, factors such as gender, teaching experience, and age are highly relevant contextual moderators.
Recommendation:
Authors should include a justification for the representativeness of their sample based on available institutional or population data and explicitly address this limitation in the manuscript. Additionally, future data collection plans to incorporate demographic diversity should be clearly integrated into the manuscript, not merely mentioned in the response letter.

Comment 18: Provide more information on participants’ demographics (e.g., gender, age, years of experience, educational background) to assess whether the sample is diverse enough for developing a valid Emotional Labor Ability Scale.

The authors' response does not address the core issue: without a demographic breakdown, the applicability of the scale across subgroups remains unknown. Furthermore, the current manuscript provides no empirical evidence that the sample is representative.

Recommendation:

The authors must clearly state in the manuscript that the generalizability of their results is limited due to the lack of demographic information and propose a concrete plan for stratified validation in future research stages.

**Thanks for the comment. Due to the correlation between the two comments mentioned above, I will integrate them and provide a unified response.**

**The demographic variable characteristics of the target group are one of the factors for verifying sample representativeness. At the same time, we need to note that there are other factors that can ensure the representativeness of the sample. For example, previous studies have shown that survey data with large sample sizes, diverse sample regions, and random sampling methods can improve sample representativeness and reliability, making research results more likely to approach the true characteristics of the population^[1]^^[2]^.**

**In the specific context of this study,The number of valid questionnaires in this study is 22.1 times the total number of questions, which is much larger than the theoretical sample threshold (37 × 10). Moreover, this study to some extent considered the geographical distribution of data sources. For the convenience of personal work and study, this study mainly randomly distributed questionnaires to Hunan Province, Xinjiang Uyghur Autonomous Region, and Jiangsu Province. Among them, Hunan Province distributed 366 questionnaires, Xinjiang Uyghur Autonomous Region distributed 158 questionnaires, and Jiangsu Province distributed 294 questionnaires. The sample size has covered the eastern, central, and western regions of China. In summary, this study demonstrated a high degree of representativeness and diversity in the research data due to its large sample size, extensive geographical coverage, and random sampling method.**

**At the same time, despite my great efforts in distributing the questionnaire, I was unable to balance the proportion of demographic variables among the survey subjects due to the limited personal abilities of the researchers. Therefore, although the scale has been formed based on solid theoretical foundations, two rounds of expert opinions, interviews with grassroots kindergarten teachers, and a large amount of questionnaire survey data, it still needs to expand the sample size and empirical research scope in the future to enhance its representativeness and applicability.**

**Based on the comment, I have made the following modifications to the paper:**

**2.2.4 Questionnaire Survey**

**The questionnaire comprises five dimensional elements and 37 items, with each question item adopting the Likert five-point scoring method to facilitate quantitative analysis. Previous studies have shown that survey data with large sample sizes, diverse sample regions, and random sampling methods can improve sample representativeness and reliability, making research results more likely to approach the true characteristics of the population (**Xu & Sun, 2025; Hao & Jin , 2020)**. Therefore, in order to improve the representativeness and diversity of the sample, This study mainly aimed to randomly distribute questionnaires to in-service kindergarten teachers in Hunan Province, Xinjiang Uygur Autonomous Region, and Jiangsu Province, with a total of 818 valid questionnaires collected. Among them, Jiangsu Province collected 294 valid questionnaires, Hunan Province collected 366 valid questionnaires, and Xinjiang Uygur Autonomous Region collected 158 valid questionnaires. The sample size has covered the eastern, central, and western regions of China. In addition, Previous studies have suggested that the number of valid questionnaires should be at least 10 times the number of items on the scale to be more reliable (He, 2025). The number of valid questionnaires in this study is 22.1 times the total number of questions, which is much larger than the theoretical sample threshold (37 × 10). In summary, this study demonstrates high representativeness and diversity with its large sample size, extensive geographical coverage, and random sampling method. After collecting valid data, use SPSS software to randomly split the data into two parts. The first 409 pieces of data were used for exploratory factor analysis (EFA), which aimed to identify the underlying factors or dimensions of the questionnaire. The other 409 pieces of data were reserved for confirmatory factor analysis (CFA), which was conducted to verify the factor structure identified in the EFA and assess the questionnaire's construct validity. Overall, this study employed several analytical methods including item analysis, exploratory factor analysis, confirmatory factor analysis, reliability and Validity analysis.**

**5. Limitations and Future Research**

**Second, although the scale has been formed based on solid theoretical foundations, two rounds of expert opinions, interviews with grassroots kindergarten teachers, and a large amount of questionnaire survey data, research results also indicate that the scale has good psychological measurement characteristics. However, the lack of demographic variable information may lead to limited universality of research results. Therefore, in the future stage of large sample surveys, we will include core demographic indicators such as age, teaching experience, education, kindergarten type, etc. for further in-depth exploration to improve the representativeness and universality of research results.**

**I have added the new literature introduced in this revision to the reference list:**

**[15]Hao Y.W., Jin Y.J.. (2020). Design of Spatial Balanced Sampling with Geographic Coordinate Information Participation. Mathematical Statistics and Management, 39 (06): 978-989. https://doi.org/10.13860/j.cnki. sltj. 2020, 1025-001.**

**[40]Xu S.C., Sun Q.. (2025). The Influence of Family Social Capital on College Students' Fertility Intention. Journal of Social Sciences, Jilin University, 65 (02): 181-195+239. https://doi.org/10.15939/j.jujsse.2025.02.sh2.**

Comment 20: Clarify whether the questionnaire includes negatively worded items that require reverse scoring and explain how these items are handled in the final scoring process.

The authors explicitly state that no reverse-scored items were included; however, this is inconsistent with the wording of several items, which are negatively phrased and logically require reverse scoring to align with the intended scale directionality (i.e., higher scores reflecting stronger emotional labor ability).

Upon review, the following items appear negatively worded and may require reverse coding:

• Item 9: "For me, the normative requirements that kindergarten teachers need to follow when expressing their emotions are not unfamiliar."

→ The double negative structure introduces ambiguity; it may function as a reverse item depending on interpretation.

• Item 17: "When interacting with young children, I can naturally show a sad emotional state to meet work needs (such as reaching empathy with young children)."

→ Agreement may indicate positive ability, but the interpretation could vary.

• Item 18: "When interacting with young children, I can naturally show an angry emotional state to meet work needs (such as expressing negation of problematic behavior in young children)."

→ Similar concerns apply regarding the positive/negative framing.

• Item 19: "When interacting with young children, I can naturally show a tense emotional state to meet work needs (such as attracting children's attention, etc.)."

→ The framing may confuse respondents without clarification.

• Item 32: "When my mood is relatively low, I can still persist in reflecting on whether my emotional expression behavior is reasonable."

→ Despite positive intent, the "low mood" condition could affect responses.

Recommendation:

To ensure scoring accuracy and transparency:

1. Review and identify all items with negative or ambiguous phrasing, especially those listed above.

2. Clearly indicate in the manuscript whether these items were reverse-coded or not. If not, discuss the rationale and implications for validity.

3. Consider rewording these items in future iterations to avoid semantic confusion (especially Item 9).

4. Include a scoring guide (e.g., a coding table) as an appendix or supplementary file to facilitate replicability and future validation studies.

Failure to handle reverse-coded items properly could distort factor structures, produce misleading total scores, and lead to misinterpretations of emotional labor profiles.

**Thanks for the comment. The above items do not require reverse coding because the higher the score of the above items, the higher their emotional labor ability.**

**Item 9: "For me, the normative requirements that kindergarten teachers need to follow when expressing their emotions are not unfamiliar."**

**Kindergarten teachers are not unfamiliar with the normative requirements that need to be followed when expressing emotions. This sentence means that kindergarten teachers are familiar with the laws of emotional labor.**

**The higher the score of this question, the more familiar the kindergarten teacher is with the rules of emotional labor and the stronger their emotional labor ability.**

**Item 17: "When interacting with young children, I can naturally show a sad emotional state to meet work needs (such as reaching empathy with young children)."**

**Item 18: "When interacting with young children, I can naturally show an angry emotional state to meet work needs (such as expressing negation of problematic behavior in young children)."**

**Item 19: "When interacting with young children, I can naturally show a tense emotional state to meet work needs (such as attracting children's attention, etc.)."**

**Firstly, I need to point out that in the context of kindergarten education, emotional states such as sadness, anger, and tension are not entirely negative. On the contrary, in different types of educational activities such as picture book storytelling and outdoor sports activities, kindergarten teachers often need to express the above different types of emotions according to specific teaching situations to achieve corresponding teaching goals. Therefore, for the group of kindergarten teachers, naturally showing emotional states such as sadness, anger, and tension in front of young children is a very normal educational behavior, and to some extent, it is also a teaching method.**

**Secondly, the above three items mainly point to the fourth dimension of emotional labor ability: the ability to apply emotional labor strategies. The ability to apply emotional labor strategies refers to the ability of kindergarten teachers to use certain emotional labor strategies according to the needs of educational contexts during the emotional labor process, including two sub dimensions: the ability to apply deep performance strategies and the ability to apply natural performance strategies. Among them, the ability to apply natural expression strategies is a higher-level ability than the ability to apply deep expression strategies. The higher the scores of the above three items, the more likely kindergarten teachers are to naturally express appropriate emotional behaviors in complex and changing educational situations to meet organizational requirements, that is, the higher their ability to apply natural expression strategies.**

**Item 32: "When my mood is relatively low, I can still persist in reflecting on whether my emotional expression behavior is reasonable."**

**This question mainly points to the fifth dimension of emotional labor ability of kindergarten teachers: emotional labor reflection ability. In this study, the dimension of emotional labor reflection ability includes three sub dimensions: reflective perseverance, the ability to reflect based on relevant theoretical knowledge, and the ability to reflect based on emotional labor experience. Among them, when my emotions are relatively low, I can still persist in reflecting on whether my emotional expression behavior is reasonable, which precisely demonstrates the reflective perseverance of kindergarten teachers. The higher the score of this question, the stronger the reflective perseverance in the emotional labor reflection ability of kindergarten teachers.**

**To make the expression easier to understand, I have changed the wording of item 9 to:**

**I am familiar with the emotional expression norms that kindergarten teachers need to follow**

Comment 21: Clarify how participant scores were handled and presented.

Although the methodology appears reasonable, the presentation of results is incomplete. The manuscript does not report descriptive statistics (means, standard deviations, ranges) for total or subscale scores. This omission limits the reader's ability to interpret the scale’s effectiveness and score distribution.

Recommendation:

The authors should report descriptive statistics (mean, SD, minimum, maximum) for total and subscale scores within the manuscript to allow readers to evaluate the scale’s distributional properties and interpretability.

**Thanks for the comment.**

**In this study, our fundamental objective is to develop and validate the psychometric characteristics of the scale (such as structural validity and reliability), and we need to ensure that all research steps serve this fundamental objective. If we add the scores of kindergarten teachers on each dimension of the scale, on the one hand, too much information would blur the focus of this study. On the other hand, since this study does not provide demographic variable information, merely adding the scores of kindergarten teachers on each dimension of the scale would have little reference value. The above is my thinking on this issue for your reference. If you still think it is necessary to add it, I can do so later.**

Inconsistency in Number of Items Reported
Observation:
In the manuscript, the authors refer to a 39-item scale, whereas the submitted version for review contains only 33 items.
Recommendation:
The authors must:
1. Clarify the final number of items on the validated scale.
2. Explain whether any items were excluded during factor analysis.
3. Include a complete, numbered list of the final items in an appendix or supplementary material to ensure transparency.

**Thanks for the comment.**

**The number of items on the scale is consistent with the description in the paper.**

**Firstly, on page 9 of the paper, we state: The questionnaire comprises five dimensional elements and 37 items。**

**Secondly, on page 12 of the paper, we write: Among them, delete the four items “E1, E2, E3, B2”, and the resulting item load values and commonality are shown in Table 3.**

**Therefore, in this study, there were a total of 33 items on the scale.**

1. Xu S.C., Sun Q.. (2025). The Influence of Family Social Capital on College Students' Fertility Intention. *Journal of Social Sciences, Jilin University*, 65 (02): 181-195+239. https://doi.org/10.15939/j.jujsse.2025.02.sh2.
2. Hao Y.W., Jin Y.J.. (2020). Design of Spatial Balanced Sampling with Geographic Coordinate Information Participation. *Mathematical Statistics and Management*, 39 (06): 978-989. https://doi.org/10.13860/j.cnki. sltj. 2020, 1025-001.
