# Supplementary material for: Development of emotional labor ability scale for kindergarten teachers
Source: PLoS One. 2025 Jun 23;20(6):e0325891. doi: 10.1371/journal.pone.0325891 (PMC12184924; doi:10.1371/journal.pone.0325891)
Supplement: Attachment — Submitted filename: Response_to_the_Reviewers_auresp_4.docx [file pone.0325891.s017.docx]

**Report**

**Additional Editor Comments** 14.05.2025

1.Clarify sampling methodology

Recommendations:
Explicitly clarify sampling as convenience-based

Thank you for your interest in the sampling method of this study. The expressions of convenience sampling and random sampling mentioned in the article are not contradictory. Now, the application relationship between convenience sampling and random sampling is explained as follows:

① Convenient Sampling for Regional Selection

Based on the researcher's existing research cooperation networks in Xinjiang Uygur Autonomous Region (western region), Hunan Province (central region), and Jiangsu Province (eastern region), we used convenience sampling to determine the sample area. This design ensures that the sample covers the three major economic geographical zones of eastern, central, and western China, thereby enhancing the geographical representativeness of the sample.

② Random sampling of teacher groups

In the selected area, we distributed questionnaires to in-service kindergarten teachers through random sampling to ensure that each teacher has an equal probability of being included in the study and avoid human selection bias.

The statement in the paper is:

Therefore, based on the researcher's existing research cooperation networks in Xinjiang Uygur Autonomous Region (western region), Hunan Province (central region), and Jiangsu Province (eastern region), we used convenience sampling to determine the sample area. This design ensures that the sample covers the three major economic geographical zones of eastern, central, and western China, thereby enhancing the geographical representativeness of the sample. In the selected area, we distributed questionnaires to in-service kindergarten teachers through random sampling to ensure that each teacher has an equal probability of being included in the study and avoid human selection bias.

2.Add demographic information
Recommendations:

Disclose available demographic data, even if imbalanced

Thank you for your attention to the sample features. This study has collected gender information from participants, including 50 male kindergarten teachers and 768 female kindergarten teachers. Based on your suggestion, we have added the following content in the relevant section of the paper:

In the valid questionnaire, there were 50 male kindergarten teachers, accounting for 6.11% of the total number, and 768 female kindergarten teachers, accounting for 93.89% of the total number.

At the same time, it should be noted that this study, as the initial validity validation stage of the scale development, was mainly based on the core objectives of psychometric assessment and did not systematically collect other demographic variables such as age and teaching experience. We acknowledge that this limitation may affect the group inference validity of the results. Based on your previous suggestion, we have made the following clarification on the limitations of our research:

Second, although the scale has been formed based on solid theoretical foundations, two rounds of expert opinions, interviews with grassroots kindergarten teachers, and a large amount of questionnaire survey data, research results also indicate that the scale has good psychological measurement characteristics. However, the lack of demographic variable information may lead to limited universality of research results. Therefore, in the future stage of large sample surveys, we will include core demographic indicators such as age, teaching experience, education, kindergarten type, etc. for further in-depth exploration to improve the representativeness and universality of research results.

3.Include descriptive statistics
Recommendations:

Mandate the inclusion of a table for descriptive statistics.

Thank you for your attention to the descriptive statistics. Based on your suggestion, I have added descriptive analysis to the paper, as follows:

According to the analysis of Descriptive Statistics (Table 8), The average values of emotional intelligence, the ability of internalizing emotional labor rules, the coordination ability in emotional labor, the reflective ability after emotional labor, and the application ability to emotional labor strategies in each dimension are 3.94, 4.05, 4.00, 3.99, and 3.97, respectively. The mean values of all dimensions are significantly higher than the theoretical median, indicating that the surveyed group of kindergarten teachers generally self evaluate to have high emotional labor ability. The standard deviation of each dimension ranges from 0.54 to 0.61, and the variance ranges from 0.30 to 0.38, indicating a relatively low degree of data dispersion.

| Table 8 Descriptive Statistics(N=818) | | | | | | |
| --- | --- | --- | --- | --- | --- | --- |
| Factor | range | minimum value | Maximum value | average value | standard deviation | variance |
| A emotional intelligence | 4.00 | 1.00 | 5.00 | 3.94 | 0.54 | 0.30 |
| B the ability of internalizing emotional labor rules | 4.00 | 1.00 | 5.00 | 4.05 | 0.59 | 0.35 |
| C the coordination ability in emotional labor | 4.00 | 1.00 | 5.00 | 4.00 | 0.60 | 0.37 |
| D the reflective ability after emotional labor | 4.00 | 1.00 | 5.00 | 3.99 | 0.57 | 0.33 |
| E the application ability to emotional labor strategies | 3.83 | 1.00 | 5.00 | 3.97 | 0.61 | 0.38 |

4.Provide complete final scale items

Recommendations:

Include the final 30-item scale in full (wording + subscales) as a table or appendix.
Justify all item deletions

①Include the final 30-item scale in full (wording + subscales) as a table or appendix：According to the suggestion, I have explained the dimensions, question items, and corresponding scoring rules in the table, as shown in the following table:

|  | Copy of the Emotional Labor Ability Scale for Kindergarten Teachers | | |
| --- | --- | --- | --- |
| dimension | | items | Scoring principle |
| Emotional intelligence | | I can judge a child's emotional state through their facial expressions | All items are scored using the Likert five point scoring method. The higher the score, the stronger the emotional labor ability of kindergarten teachers in the corresponding dimension |
|  |  | I can judge a child's emotional state by their tone of speech |  |
|  |  | I can judge a child's emotional state through their body movements |  |
|  |  | I can judge a child's emotional state through their physiological arousal (shortness of breath, tremors, etc.) |  |
|  |  | I can judge the compound emotions of young children (such as anger, restlessness, surprise, etc.) by observing their words and expressions |  |
|  |  | I can understand why young children feel happy |  |
|  |  | I can understand why young children feel scared |  |
|  |  | I can understand why young children feel angry |  |
| The Ability of Internalizing Emotional Labor Rrules | | I am familiar with the emotional expression norms that kindergarten teachers need to follow |  |
|  |  | I strongly agree with the normative requirements that kindergarten teachers should follow when expressing emotions, such as gentleness, kindness, enthusiasm, etc |  |
|  |  | My attitude towards the normative requirements that kindergarten teachers should follow in expressing their emotions and behaviors is relatively stable |  |
|  |  | I firmly believe that the emotional expression behavior of kindergarten teachers must follow certain normative requirements |  |
|  |  | I strongly advocate that kindergarten teachers must follow certain normative requirements when expressing their emotions |  |
| The Application Ability To Emotional Labor Strategies | | When interacting with young children, if I need to show excitement, I can feel it from the bottom of my heart |  |
|  |  | When interacting with young children, if I need to show enthusiasm, I can feel it from the bottom of my heart |  |
|  |  | When interacting with young children, if I need to show a relaxed mood, I can feel it from the bottom of my heart |  |
|  |  | When interacting with young children, I can naturally show a sad emotional state to meet work needs (such as reaching empathy with young children) |  |
|  |  | When interacting with young children, I can naturally show an angry emotional state to meet work needs (such as expressing negation of problematic behavior in young children) |  |
|  |  | When interacting with young children, I can naturally show a tense emotional state to meet work needs (such as attracting children's attention, etc.) |  |
| The Coordination Ability In Emotional Labor | | When dealing with emotional events, I can still ensure the smooth progress of children's morning activities |  |
|  |  | When dealing with emotional events, I can still ensure the smooth progress of collective teaching activities for young children |  |
|  |  | When dealing with emotional events, I can still ensure the smooth progress of children's daily activities (such as eating, drinking, washing, toileting, sleeping, leaving the kindergarten, etc.) |  |
|  |  | When dealing with emotional events, I can still ensure the smooth progress of activities in the children's activity area |  |
|  |  | When dealing with emotional events, I can still ensure the smooth progress of outdoor activities for young children |  |
|  |  | When dealing with emotional events, I can still ensure the smooth progress of children's activities outside the kindergarten |  |
| The Reflective Ability After Emotional Labor | | I can reflect on the rationality of my emotional expression behavior based on my experience of emotional interaction with young children |  |
|  |  | I can reflect on the rationality of my emotional expression behavior based on my own emotional expression experience |  |
|  |  | I can reflect on whether my emotional expression behavior contributes to the development of children's emotional intelligence based on relevant theoretical knowledge |  |
|  |  | I can reflect on whether my emotional expression behavior helps meet the emotional needs of young children based on relevant theoretical knowledge |  |
|  |  | I can reflect on whether my emotional expression behavior meets the emotional requirements of kindergarten regulations based on relevant theoretical knowledge |  |
|  |  | I can persistently reflect on whether my emotional expression and behavior are reasonable |  |
|  |  | When my mood is relatively low, I can still persist in reflecting on whether my emotional expression behavior is reasonable |  |
|  |  | To make my emotional expression more in line with expectations, I am able to actively reflect on my emotional expression behavior on a continuous basis |  |

②Justify all item deletions：

I have explained the rationality of deleting items in the corresponding section of the paper, as follows:

Using principal component analysis and maximum variance method to extract common factors, and then deleting items with factor load value less than 0.5, commonality less than 0.4, multiple loads with similar load values, or improper factor classification, a total of “E1, E2, E3, B2” items were deleted, with 33 items remaining.
